# Molecular Mechanisms of Oil and Saponin Accumulation and the Regulation of Carbohydrate Metabolism in *Sapindus mukorossi* Fruit

**DOI:** 10.3390/plants15142173

**Published:** 2026-07-15

**Authors:** Xiao Zhou, Changzhu Li, Yunzhu Chen, Yan Yang, Qiang Liu, Wenbin Zeng, Luhong Zhang, Lijuan Jiang, Zhihong Xiao, Yuena Ji, Li Li, Hao Wang, Peiwang Li, Jingzhen Chen

**Affiliations:** 1 State Key Laboratory of Woody Oil Resources Utilization, Hunan Academy of Forestry, Changsha 410018, China; zx924647234@163.com (X.Z.);; 2 College of Life Science and Technology, Central South University of Forestry and Technology, Changsha 410004, China; 3 Key Laboratory of National Forestry and Grassland Administration on Utilization Science for Southern Woody Oilseed, Hunan Academy of Forestry, Changsha 410018, China

**Keywords:** soapberry, morphological dynamics, triterpenoid biosynthesis, triacylglycerol biosynthesis, carbon flux, multi-omics analysis

## Abstract

*Sapindus mukorossi*, known as soapberry, is a multipurpose woody species with oil-rich kernels and saponin-rich pulp, and its kernel oil and pulp saponins have important applications in oleochemicals, detergents, cosmetics, pharmaceuticals, and bioenergy. However, the mechanism by which carbohydrate-derived carbon is differentially allocated to kernel oil biosynthesis and pulp saponin biosynthesis within the same fruit remains unclear. In this study, we integrated morphological, physiological, metabolomic, and transcriptomic analyses to investigate the developmental accumulation patterns of oil and saponins and their relationship with carbohydrate metabolism in *S. mukorossi* fruit. The fruit reached maturity at approximately 130 days after flowering and was divided into four developmental stages: slow growth, rapid expansion, color transition, and maturation. Kernel oil accumulated in a typical S-shaped pattern and increased rapidly during the middle-to-late developmental stages, whereas pulp saponins increased continuously before maturity and remained stable. At maturity, soluble sugars mainly accumulated in the pulp, while starch was relatively enriched in the kernels, indicating different carbohydrate storage and utilization patterns between the two tissues. Integrated transcriptomic and metabolomic analyses revealed tissue-specific metabolic regulation: lipid metabolism was preferentially enriched in kernels, whereas terpenoid and secondary metabolic pathways were more active in pulp. Several genes related to fatty acid assembly and oil accumulation, including *SmACC*, *SmLACS*, *SmGPAT*, and *SmLPAT*, showed kernel-biased expression, while genes involved in the mevalonate (MVA)-dependent triterpenoid saponin pathway, including *SmACCT*, *SmMVK*, *SmIDI*, *SmSQE*, and *SmLUP2*, were closely associated with pulp saponin accumulation. Hormone-related transcription factors, such as GRAS and BES1 in kernels and MYC2 in pulp, may contribute to the coordination between carbohydrate metabolism and oil or saponin biosynthesis. Overall, these findings suggest that oil and saponin accumulation in *S. mukorossi* fruit are regulated by distinct tissue-specific metabolic programs closely linked to carbohydrate metabolism. This study provides new insights into carbon allocation between kernel oil and pulp saponin biosynthesis and identifies candidate pathways and genes for the genetic improvement and resource utilization of *S. mukorossi*.

## 1. Introduction

*Sapindus mukorossi* Gaertn., commonly known as soapberry or wash nut, is a deciduous tree belonging to the genus *Sapindus* in the family Sapindaceae. It is widely distributed in eastern, southern, and southwestern China [1,2]. The kernels exhibit a high oil content of up to 42.70%, while the fruit pulp (exocarp and mesocarp) contains saponins up to 33.91% [3]. These two tissue-specific products have considerable economic value and are widely used in detergents, pharmaceuticals, cosmetics, and bioenergy [4]. Current research on *S. mukorossi* primarily focuses on the extraction techniques and content determination of saponins and oils, whereas their biosynthetic mechanisms and regulatory networks remain insufficiently understood. As the two major carbon-rich products in soapberry fruit, kernel oil and pulp saponins are derived from primary carbohydrate metabolism and share glucose-derived carbon sources and acetyl-CoA-related precursors. Therefore, the differential allocation of carbon between the kernel and pulp is not only related to fruit quality formation, but also represents an important biological issue involving the coordination between primary carbon metabolism, storage oil biosynthesis, and specialized triterpenoid saponin biosynthesis within the same fruit. However, little is known about how carbohydrate-derived carbon is partitioned between these two sink tissues during fruit development and how this process is transcriptionally regulated.

Carbohydrate metabolism is central to plant energy metabolism and carbon metabolism. Through the decomposition and transformation of sugars, it provides the carbon skeletons, energy, including ATP and NADPH, and key precursors for the synthesis of oils and saponins [5,6]. The accumulation of oils and saponins in soapberry is a complex process involving carbon supply, precursor conversion, enzyme activity, gene expression, and signal regulation. Carbohydrate metabolism may influence the biosynthesis of saponins and oils through precursor supply, regulation of key genes, and integration with signaling networks [7]. For instance, sucrose, the primary transport form of photosynthetically fixed carbon, is loaded, transported through the phloem, and unloaded into different fruit tissues [8,9,10]. This process determines the spatiotemporal distribution of carbon sources and may further affect downstream metabolic pathways through sugar signaling and energy metabolism.

Previous studies have shown that carbohydrate metabolism and sugar-related regulatory networks can influence both storage metabolism and specialized metabolism. To understand the potential regulatory role of carbohydrate metabolism in secondary metabolism, Zhang M.J. et al. [6] applied exogenous sugars (sucrose, glucose/fructose) to the leaves of *Cyclocarya paliurus*. Their results showed that exogenous sugars promoted the accumulation of soluble sugars and starch, increased the activity of enzymes related to carbohydrate metabolism, and enhanced the accumulation of flavonoids and terpenoids. Wang H. et al. [11] demonstrated that the soybean Dof-type transcription factors *GmDof4* and *GmDof11* increased oil content in transgenic *Arabidopsis thaliana* by activating genes encoding acetyl-CoA carboxylase and long-chain acyl-CoA synthetase, reducing the expression of the seed storage protein gene CRA1, and redirecting energy flux toward fatty acid and oil synthesis. These studies suggest that carbohydrate metabolism can participate in regulating both oil accumulation and specialized metabolite biosynthesis. However, how carbohydrate metabolism coordinates the differential accumulation of oil and saponins between distinct fruit tissues remains unclear.

Based on the changes in oil, saponins and carbohydrate content during soapberry fruit development, this study integrated physiological, transcriptomic and metabolomic analyses to investigate the relationship between carbohydrate metabolism and the tissue-specific accumulation of kernel oil and pulp saponins. We hypothesized that carbohydrate metabolism may act as a central metabolic hub that differentially allocates carbon toward fatty acid and oil biosynthesis in kernels and MVA-dependent triterpenoid saponin biosynthesis in pulp through tissue-specific gene expression and hormone-related regulatory networks. Accordingly, this study aimed to clarify the developmental accumulation patterns of oil and saponins, identify key metabolic pathways and candidate genes involved in carbon allocation, and construct a regulatory framework linking carbohydrate metabolism with oil and saponin biosynthesis. The findings are expected to provide a theoretical basis for targeted breeding, variety improvement, and cultivation management of *S. mukorossi*.

## 2. Results

### 2.1. Fruit Morphological Development Characteristics

**Figure 1:**
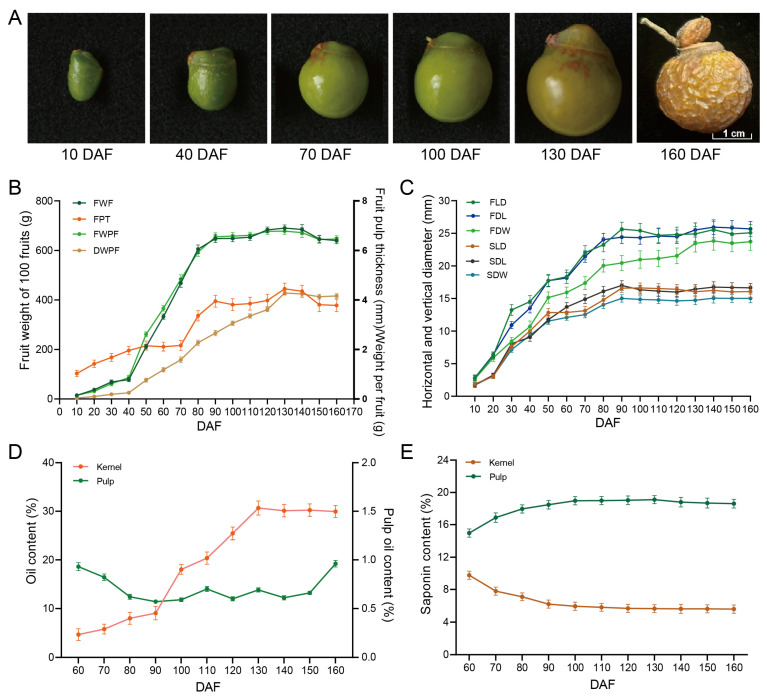
Development patterns of *S. mukorossi* fruits and accumulation dynamics of oils and saponins. Note: (**A**): Morphology of *S. mukorossi* fruits at 10, 40, 70, 100, 130, and 160 DAF. (**B**): Changes in fresh weight of 100 fruits and pulp thickness at different development stages of *S. mukorossi* fruit. (**C**): Phenotypic changes in *S. mukorossi* fruit and seed at different developmental stages. (**D**): Changes in oil content in the kernel and pulp of *S. mukorossi* fruit (dried powder samples) at different developmental stages. (**E**): Changes in saponin content of kernel and pulp of *S. mukorossi* fruit at different developmental stages. FWF: fresh weight of 100 fruits, PT: pulp thickness, FWPF: fresh weight per fruit, DWPF: dry weight per fruit, FLD: fruit longitudinal diameter, FDL: maximum transverse diameter, FDW: minimum transverse diameter, SLD: seed longitudinal diameter, SDL: maximum transverse diameter, and SDW: minimum transverse diameter.

Phenological observations of *S. mukorossi* revealed that in the study area during 2023, inflorescences and flower buds began to develop in late April, with the initial fruit stage occurring in mid-June. Based on changes in fruit size and weight, the fruit development process was divided into the following stages, each with distinct characteristics: (1) Slow growth period (10~40 DAF): fruits were dark green, with slow increases in volume and weight, kernels consisted of liquid endosperm. (2) Rapid expansion period (40~90 DAF): the peel color changed from dark green to light green, with rapid increases in volume and weight; kernels developed their definite shape. (3) Color change period (90~130 DAF): the peel began to yellow, transitioning from light green to yellowish-green, volume and weight showed no significant changes compared to the previous period, kernels reached maturity. (4) Maturation period (130~160 DAF): fruit volume and weight largely stabilized, the smooth, glossy peel turned from yellowish-green to orange-yellow (Figure 1A–C).

The pulp thickness exhibited a characteristic double sigmoidal (S-shaped) growth pattern (Figure 1A). It increased slowly from 10 to 70 DAF, rapidly from 70 to 90 DAF, and then stabilized with minor fluctuations from 90 to 160 DAF, reaching its maximum thickness at 130 DAF. The change trend of fresh weight per fruit of *S. mukorossi* is basically consistent with that of fresh weight of 100 fruits. Dry weight per fruit increases slowly from 10 to 40 DAF, grows rapidly from 40 to 130 DAF, and reaches its maximum at 130 DAF. The morphology of the soapberry fruits and seeds developed synchronously, both showing an overall sigmoidal (S-shaped) upward trend. Fruit morphology remained largely unchanged during the maturation period, while seed morphology stabilized after the color change period (Figure 1C).

### 2.2. Dynamics of Oil and Saponin Accumulation

Phenological observations confirmed that solid kernels began to develop in *S. mukorossi* fruits at 60 DAF. Consequently, the oil content in kernels and pulp, as well as saponin content, were measured from 60 to 160 DAF. The results revealed that oil accumulation in the kernels followed a typical triphasic “S-shaped” pattern: an initial slow accumulation phase (60~90 DAF) during which the oil content increased from 4.7% to 9.1%, a rapid accumulation phase (90~130 DAF) where the oil content surged to 30.7%, and a maturation and stabilization phase (130~160 DAF) where it remained at its peak level (Figure 1D). In contrast, the oil content in the pulp exhibited a different accumulation pattern, showing a double “W-shaped” fluctuating trend. An initial peak (0.6%) occurred during the early developmental stage (60~80 DAF), followed by a secondary peak (0.8%) in the mid-stage (100~120 DAF), ultimately reaching the highest value of 1.0% at maturity (160 DAF) (Figure 1D).

The saponin content in the kernels and pulp of soapberry fruits varied significantly across different developmental stages, demonstrating notable tissue specificity (Figure 1E). Pulp saponins displayed a gradual upward accumulation pattern: rapid accumulation from 10~70 DAF, with content rising from 9.9% to 17.9%, slow accumulation from 70~130 DAF, increasing to the maximum of 19.10%, and stabilization from 130~160 DAF, with no significant changes in pulp saponin content (Figure 1E). Conversely, the trend in kernel saponin content was opposite. At the initial stage of kernel formation (60 DAF), kernel saponin content was highest at 9.8%, sharply decreased to 5.9% by 100 DAF, and subsequently remained largely unchanged.

Therefore, oil is the primary metabolite in the *S. mukorossi* kernels, whereas saponins are the primary metabolites in the soapberry pulp. Based on the developmental pattern of *S. mukorossi* fruits (four major developmental stages) and the accumulation characteristics of their main internal components (oil and saponins), kernels and pulp from three key periods, 70 DAF (S1), 100 DAF (S2), and 130 DAF (S3), were selected for subsequent comparative studies.

### 2.3. Carbohydrate Content Changes

**Figure 2:**
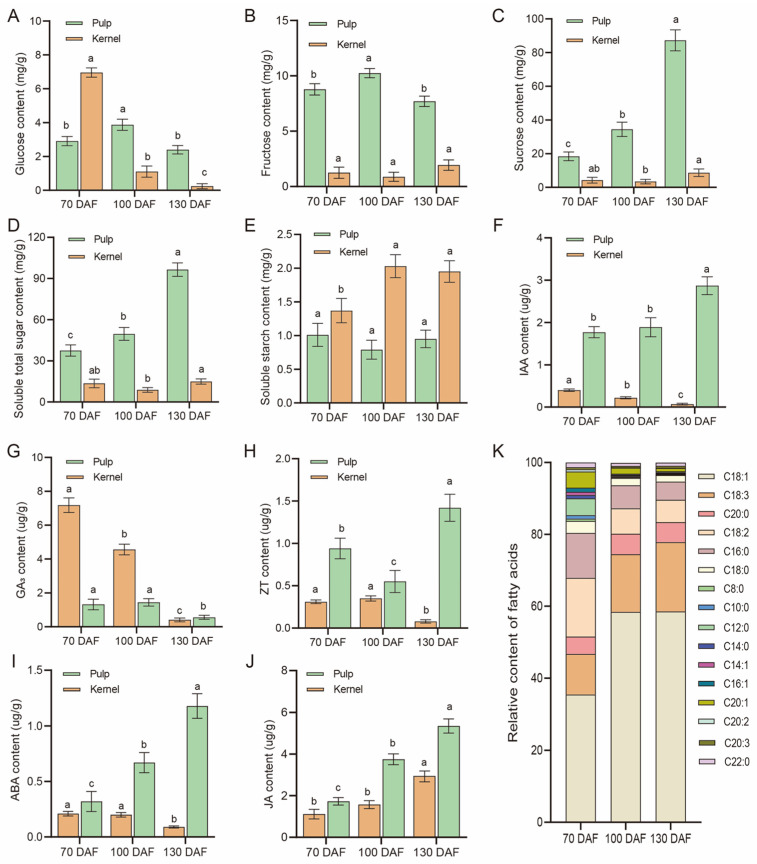
Changes in the contents of soluble sugars and endogenous hormones in the kernel and pulp of *S. mukorossi* fruit at different developmental stages. (**A**–**E**): Representing glucose content, fructose content, sucrose content, soluble total sugar content, and soluble starch content in the *S. mukorossi* kernel and pulp, respectively. (**F**–**J**): Representing IAA content, GA3 content, ZT content, ABA content and JA content in the *S. mukorossi* kernel and pulp, respectively. (**K**): *S. mukorossi* Kernel fatty acid composition. C8:0: caprylic acid, C10:0: capric acid, C12:0: lauric acid, C14:0: myristic acid, C14:1: myristoleic acid, C16:0: palmitic acid, C16:1: palmitoleic acid, C18:0: stearic acid, C18:1: oleic acid, C18:2: linoleic acid, C18:3: linolenic acid, C20:0: arachidic acid, C20:1: gondoic acid, C20:2: eicosadienoic acid, C20:3: eicosatrienoic acid, C22:0: behenic acid. Different lowercase letters indicate significant differences among developmental stages within the same tissue at *p* < 0.05.

Glucose content in both the pulp and kernels of *S. mukorossi* generally displayed a decreasing trend. Kernel glucose was highest at 7.0 mg/g at 70 DAF and lowest at 0.3 mg/g at 130 DAF. Pulp glucose content first increased and then decreased, peaking at 3.9 mg/g at 100 DAF and reaching its lowest value of 2.4 mg/g at 130 DAF (Figure 2A). In contrast, fructose content in the kernels showed an overall upward trend, reaching its maximum of 1.9 mg/g at 130 DAF. Pulp fructose content rose initially and then declined, with a peak of 10.2 mg/g at 100 DAF, which was significantly higher than the content in the kernels (Figure 2B). Sucrose content in both pulp and kernels exhibited an increasing trend. The variation in kernel sucrose content was relatively small, rising to a maximum of 8.9 mg/g at 130 DAF. In comparison, pulp sucrose content changed more substantially, reaching 87.3 mg/g at 130 DAF, significantly higher than that in the kernels (Figure 2C). Total soluble sugar content in the kernels first decreased and then increased, with a relatively small amplitude of change, and reached its highest level of 14.9 mg/g at 130 DAF. Pulp total soluble sugar content showed a consistent increasing trend, peaking at 96.5 mg/g at 130 DAF, significantly higher than that in the kernels (Figure 2D). Soluble starch content in the kernels first increased and then decreased, reaching its highest value of 2.1 mg/g at 100 DAF, followed by a slight decrease to 2.0 mg/g at 130 DAF. Conversely, the pulp exhibited the opposite pattern, decreasing first and then increasing, with the highest content of 1.0 mg/g at 70 DAF. Moreover, the soluble starch content in the kernels remained consistently higher than that in the pulp across all three developmental stages (Figure 2E). In summary, in mature *S. mukorossi* fruits, the contents of glucose, sucrose, and total soluble sugars in the pulp were substantially higher than those in the kernels, while the starch content in the kernels was significantly greater than that in the pulp.

### 2.4. Endogenous Hormone Content Changes

The IAA content in the *S. mukorossi* kernels showed a decreasing trend at 70, 100, and 130 DAF, with the highest value of 0.4 μg/g observed at 70 DAF. In contrast, the IAA content in the pulp exhibited an increasing trend, reaching its peak of 2.9 μg/g at 130 DAF. Furthermore, the IAA content in the pulp was higher than that in the kernels across all three developmental stages (Figure 2F). Both the kernels and pulp displayed a decreasing trend in GA_3_ content during these three stages, with the highest levels recorded at 70 DAF at 0.4 μg/g in the kernels and 0.6 μg/g in the pulp (Figure 2G). The ZT content in the kernels initially increased and then decreased, peaking at 0.6 μg/g at 100 DAF. Conversely, the ZT content in the pulp showed the opposite trend, reaching its maximum of 1.4 μg/g at 130 DAF. Additionally, the ZT content in the pulp was consistently higher than that in the kernels at all three time points (Figure 2H). The ABA content in the kernels demonstrated a declining trend, with the highest value of 0.2 μg/g at 70 DAF. In contrast, the ABA content in the pulp showed an increasing trend, peaking at 1.2 μg/g at 130 DAF. Similarly, the ABA content in the pulp was higher than that in the kernels throughout the three stages (Figure 2I). Both the kernels and pulp exhibited an increasing trend in JA content, with the highest levels observed at 130 DAF at 3.0 μg/g in the kernels and 5.4 μg/g in the pulp (Figure 2J).

### 2.5. Fatty Acid Composition and Dynamics in Kernels

Gas chromatography (GC) analysis detected a total of 20 fatty acids in *S. mukorossi* kernels at 70, 100, and 130 DAF. Using a threshold of 1.0% relative content for classification, six were identified as major fatty acids while the remaining fourteen were categorized as minor. At fruit maturity (130 DAF), their respective relative contents reached 96.2% and 3.7% (Figure 2K). The major fatty acids included C18:1, C18:3, C20:0, C18:2, C16:0, and C18:0, with C18:1 and C18:3 being the predominant components. Their respective proportions at fruit maturity were 58.5% and 19.3%.

### 2.6. Metabolomics Analysis of Fruit Pulp

**Figure 3:**
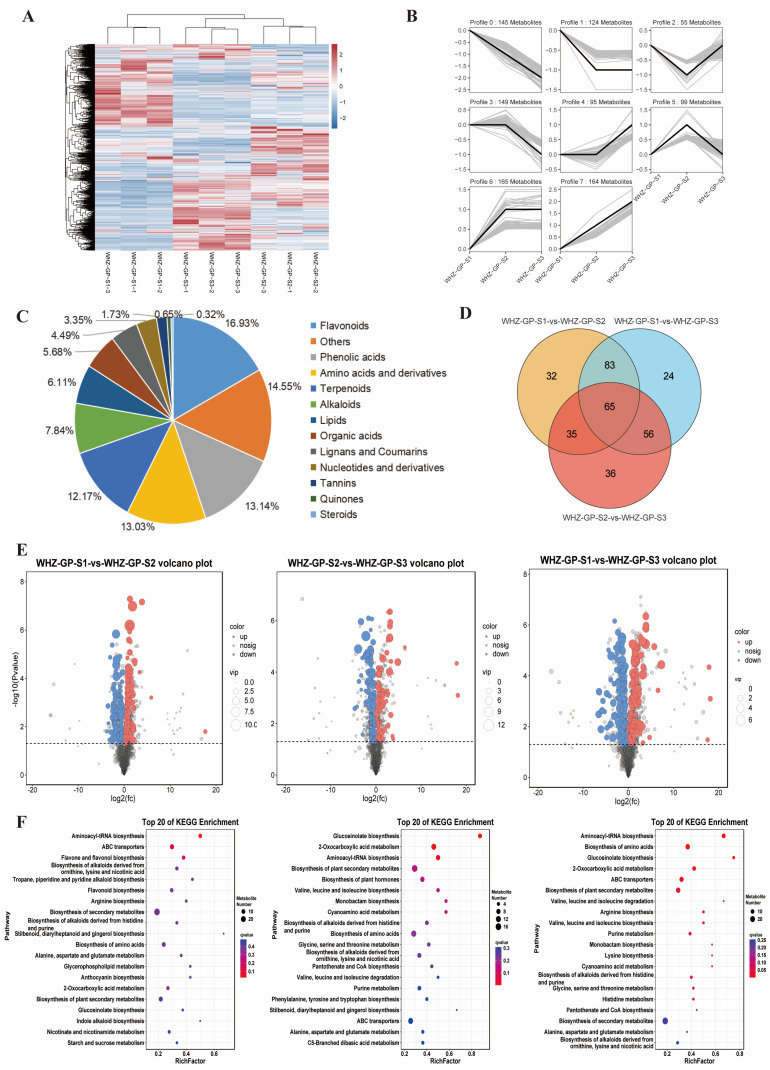
Metabolite statistics and KEGG enrichment analysis in *S. mukorossi* pulp. Note: (**A**): Metabolic profile of *S. mukorossi* pulp. (**B**): Trend analysis of DEMs in pulp. (**C**): Classification and statistics of metabolites in pulp. (**D**): Venn diagram of DEMs in pulp. (**E**): Statistics of up- and down-regulation of pulp DEMs. (**F**): DEMs KEGG enrichment analysis in pulp.

*S. mukorossi* pulp is rich in saponins, and its extracts possess multiple biological activities. To understand the changes in pulp metabolites at different developmental stages of *S. mukorossi* fruit, pulps from 70 DAF (GP-S1), 100 DAF (GP-S2), and 130 DAF (GP-S3) were selected for widely targeted LC-MS/MS metabolomic determination and analysis. A total of 1849 metabolites were detected across the three pulp stages, with 1011 detected in positive ion mode and 838 detected in negative ion mode (Figure 3A, Table S1). The dynamic patterns of metabolites were clustered into 8 categories. Among these, Profile 5 (stage-specific fluctuation type) and Profile 7 (continuous accumulation type) were dominant, containing the highest numbers of metabolites, with 165 and 164 metabolites, respectively (Figure 3B). The metabolites were predominantly Flavonoids (313), Phenolic acids (243), Amino acids and derivatives (241), and Terpenoids (225), accounting for 16.9%, 13.1%, 13.0%, and 12.2% of the total, respectively. In contrast, Quinones (12) and Steroids (6) were less abundant, accounting for only 0.65% and 0.32% of the total, respectively (Figure 3C). Comparative analysis of differential metabolites showed that the comparisons GP-S1-vs-GP-S2, GP-S1-vs-GP-S3, and GP-S2-vs-GP-S3 identified 215, 228, and 192 differential metabolites respectively, with 148, 121, and 100 co-expressed metabolites, respectively. Specifically, 32, 24, and 36 metabolites were uniquely expressed in GP-S1, GP-S2, and GP-S3 respectively, while 65 metabolites were commonly expressed across all three stages (Figure 3D).

KEGG annotation and metabolic pathway enrichment were performed on all metabolites. The results indicated that 1004 out of the 1849 metabolites were annotated and categorized into 5 major categories and 19 subcategories. The categories with the highest number of enriched metabolites were Global and overview maps (330), Amino acid metabolism (123), Biosynthesis of other secondary metabolites (96), Chemical structure transformation maps (91), and Carbohydrate metabolism (88) (Figure S1). In the comparisons GP-S1-vs-GP-S2, GP-S1-vs-GP-S3, and GP-S2-vs-GP-S3, the numbers of up-regulated differential metabolites were 112, 103, and 75, respectively, while the numbers of down-regulated differential metabolites (DEMs) were 103, 125, and 117, respectively (Figure 3E). KEGG enrichment analysis of differential metabolites revealed that pathways such as Biosynthesis of secondary metabolites, ABC transporters, Biosynthesis of plant secondary metabolites, Biosynthesis of amino acids, and Aminoacyl-tRNA biosynthesis were enriched with a relatively high number of differential metabolites. This indicates that secondary metabolism in the pulp is relatively vigorous, which may effectively enhance resistance to pests and diseases and provide a strong guarantee for seed development and maturation (Figure 3F).

### 2.7. RNA-Seq Analysis

#### 2.7.1. Transcriptome Sequencing and Mapping

To investigate the transcriptional changes associated with oil accumulation in kernels and saponin accumulation in pulp during fruit development, RNA-seq analysis was performed using 18 samples of *S. mukorossi* kernels and pulp collected at 70, 100, and 130 DAF. After sequencing, raw data were filtered, resulting in 407,463,420 (99.38%) and 490,201,120 (99.67%) high-quality clean reads obtained from the kernel and pulp samples, respectively. The high quality of the filtered data provides a reliable foundation for subsequent data analysis.

Using the published *S. mukorossi* whole genome sequence available on NCBI, the filtered clean reads (optimized by rRNA removal) were mapped to the 30,068 genes in the genome. The results showed that for the kernel samples across the three stages (9 samples in total), reads were detected in 20,146 genes, accounting for 67.00% of the annotated genes. Specifically, 18,034, 14,778, and 14,375 genes were detected at the ZR-S1, ZR-S2, and ZR-S3 stages, respectively. For the pulp samples across the three stages (9 samples in total), reads were detected in 21,924 genes, accounting for 72.91% of the annotated genes. Specifically, 16,098, 15,990, and 15,092 genes were detected at the GP-S1, GP-S2, and GP-S3 stages, respectively. A total of 3391 and 2543 novel genes were identified in the kernel and pulp samples, respectively. These novel genes were included in subsequent differential expression gene analysis (Table 1).

#### 2.7.2. Transcriptome Annotation and Expression Pattern Clustering

To obtain functional information for the expressed genes, alignment of the *S. mukorossi* kernel and pulp Clean Data with the NCBI non-redundant protein (NR) database revealed that a total of 28,221 unigenes were annotated in the kernels, with an annotation rate of 84.35%. Specifically, 17,658, 14,642, and 14,470 genes were annotated at the ZR-S1, ZR-S2, and ZR-S3 stages, respectively. In the pulp, a total of 28,802 unigenes were annotated, representing an annotation rate of 85.15%, with 19,416, 19,453, and 18,121 genes annotated at the GP-S1, GP-S2, and GP-S3 stages, respectively.

**Figure 4:**
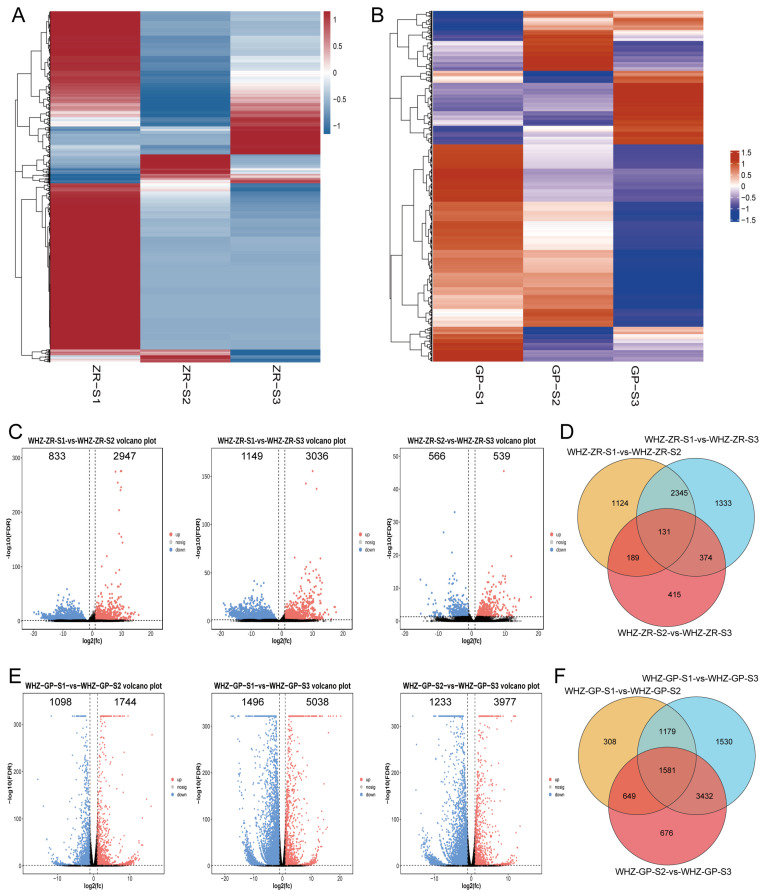
Transcriptome profiling and DEG analysis in *S. mukorossi* kernel and pulp. Note: (**A**): Gene expression profile of kernels. (**B**): Gene expression profile of pulp. (**C**): Statistics of up- and down-regulated DEGs in kernels. (**D**): Venn diagram of DEGs in kernels. (**E**): Statistics of up- and down-regulated DEGs in pulp. (**F**): Venn diagram of DEGs in pulp.

Gene expression levels in each sample were quantified using RSEM and normalized via the FPKM method for the kernel and pulp transcriptomes soapberry. To further characterize the overall transcriptional dynamics during fruit development, expressed genes were clustered according to their expression patterns across the three developmental stages. Based on Pearson correlation coefficients, both kernels and pulp were clustered into 6 distinct categories. The highest number of highly expressed genes was observed at the 70 DAF stage (ZR-S1 and GP-S1) in both tissues, suggesting that early fruit development was associated with more active transcriptional regulation. (Figure 4A,B). In the kernels, Cluster 5 contained the largest number of genes (10,693), exhibiting a continuously decreasing expression trend from 70 to 130 DAF, while Cluster 2 had the fewest genes (1873), showing an inverted “V”-shaped pattern (Figure S2). In the pulp, Cluster 4 comprised the most genes (9345), demonstrating an upward trend, whereas Cluster 5 contained the fewest genes (1763), also displaying an inverted “V”-shaped pattern (Figure S3). These expression patterns indicate that kernels and pulp underwent distinct transcriptional changes during fruit development, which may be related to their different metabolic functions.

#### 2.7.3. Transcriptome DEGs Statistical Analysis

DEGs were screened using DESeq2 (v1.34.0) software with the criteria of |log2(Fold Change)| > 1 and FDR < 0.05. A total of 5911 and 9355 DEGs were identified in the kernels and pulp of *S. mukorossi* across three developmental stages, respectively, with the number of DEGs in the pulp being substantially higher than that in the kernels. This result indicates that pulp exhibited more extensive transcriptional changes than kernels during fruit development.

DEGs analysis revealed that the comparisons of ZR-S1-vs-ZR-S2, ZR-S1-vs-ZR-S3, and ZR-S2-vs-ZR-S3 identified 3760, 4183, and 1109 DEGs in kernels, respectively. Among them, the numbers of up-regulated DEGs were 829, 1150, and 569, while the numbers of down-regulated DEGs were 2960, 3033, and 540, respectively (Figure 4C). The larger number of DEGs in the comparisons involving ZR-S1 suggests that the transition from the early stage to later stages was accompanied by marked transcriptional reprogramming in kernels. Among the 5911 total kernel DEGs, 1124, 1333, and 415 were uniquely differentially expressed in ZR-S1, ZR-S2, and ZR-S3, respectively, while 131 DEGs were commonly differentially expressed across all three stages. The numbers of DEGs commonly shared between pairwise comparisons were 2345 for ZR-S1-vs-ZR-S2, 374 for ZR-S1-vs-ZR-S3, and 189 for ZR-S2-vs-ZR-S3 (Figure 4D).

In the pulp comparisons, GP-S1-vs-GP-S2, GP-S1-vs-GP-S3, and GP-S2-vs-GP-S3 contained 3717, 7722, and 6338 DEGs, respectively. Among these, the up-regulated DEGs numbered 1406, 1662, and 1400, respectively, while the down-regulated DEGs numbered 2311, 6060, and 4938, respectively (Figure 4E). The number of down-regulated DEGs was substantially higher than that of up-regulated DEGs, especially in the GP-S1-vs-GP-S3 and GP-S2-vs-GP-S3 comparisons. In addition, Venn diagrams showed that GP-S1, GP-S2, and GP-S3 had 308, 1530, and 676 uniquely differentially expressed DEGs, respectively, with 1581 DEGs common to all three (Figure 4D). Furthermore, the numbers of common DEGs between the comparison pairs GP-S1-vs-GP-S2, GP-S1-vs-GP-S3, and GP-S2-vs-GP-S3 were 1179, 3432, and 649, respectively (Figure 4F). Together, these results indicate that pulp experienced stronger transcriptional changes than kernels, particularly during the transition toward the late developmental stage.

#### 2.7.4. Transcriptome DEGs Functional Annotations

All DEGs screened from the transcriptomes of *S. mukorossi* kernels and pulp were subjected to GO annotation and KEGG metabolic pathway enrichment to clarify their potential biological functions. GO functional classification revealed that all DEGs from both kernels and pulp were categorized into 3 main categories (level 1) and 49 subcategories (level 2) (Figure S4A). Among the 3 main categories, the highest number of DEGs were annotated to Biological Process (BP), followed by Molecular Function (MF), while Cellular Component (CC) contained the fewest annotated DEGs. Furthermore, the number of down-regulated DEGs was greater than that of up-regulated DEGs across these categories. Within BP, Cellular process and Metabolic process were the most significantly enriched terms, containing the largest numbers of both up-regulated and down-regulated DEGs. In MF, Binding was the term with the highest number of enriched DEGs, while in CC, Cellular anatomical entity was the most prominent. These results suggest that metabolic regulation was a major component of transcriptional changes during *S. mukorossi* fruit development.

KEGG pathway enrichment analysis of all kernel and pulp DEGs classified them into 5 major Class A categories and 19 Class B subcategories, involving 133 and 136 metabolic pathways, respectively (Figure S4B). Among the 5 major classes, Metabolism contained the highest number of DEGs for both tissues, followed by Genetic Information Processing. For kernels, the fewest DEGs were annotated to Organismal Systems, whereas for pulp, the fewest were annotated to Cellular Processes. Within the Metabolism category, Global and overview maps and Carbohydrate metabolism were the pathways with the highest number of enriched DEGs. This result indicates that carbohydrate metabolism was actively regulated in both kernels and pulp during fruit development.

The number of KEGG enriched metabolic pathways for the kernel comparisons ZR-S1-vs-ZR-S2, ZR-S1-vs-ZR-S3, and ZR-S2-vs-ZR-S3 was 126, 131, and 107, involving 1234, 1409, and 482 genes, respectively. For the pulp comparisons (GP-S1-vs-GP-S2, GP-S1-vs-GP-S3, GP-S2-vs-GP-S3), the number of enriched pathways was 121, 133, and 131, involving 2842, 6534, and 5210 DEGs, respectively. In the kernels, 62, 48, and 25 DEGs were annotated to Lipid metabolism in the ZR-S1-vs-ZR-S2, ZR-S1-vs-ZR-S3, and ZR-S2-vs-ZR-S3 comparisons, respectively. In the pulp, DEGs related to saponin biosynthesis were classified under Metabolism of terpenoids and polyketides. This category was enriched with 49, 85, and 82 DEGs in the GP-S1-vs-GP-S2, GP-S1-vs-GP-S3, and GP-S2-vs-GP-S3 comparisons, respectively (Figure S4B). These results highlight lipid metabolism in kernels and terpenoid-related metabolism in pulp as key pathways associated with oil and saponin accumulation, respectively, while carbohydrate metabolism may provide important precursors and energy for both processes.

### 2.8. Identification and Expression Profiling of DEGs Involved in Triterpenoid Saponin Biosynthesis in S. mukorossi

Saponins in the pulp of *S. mukorossi* are mainly pentacyclic triterpenoid saponins, especially oleanane-type saponins. To clarify the transcriptional basis of pulp saponin accumulation, DEGs involved in triterpenoid saponin biosynthesis were grouped into three functional modules: MVA and MEP/DOXP pathways, triterpenoid skeleton formation, and downstream oxidation and glycosylation modification.

In the precursor supply module, most DEGs associated with the MVA pathway, including *SmACCT*, *SmHMGS*, *SmHMG*, *SmMVK*, *SmPMK*, *SmMVD*, and *SmIDI*, showed higher expression levels at 70 and 100 DAF than at 130 DAF. This expression pattern was generally consistent with the rapid and subsequent slow accumulation phases of pulp saponins. By contrast, several DEGs assigned to the MEP/DOXP pathway, such as *SmDXR* and *SmISPH*, showed relatively low expression during the early rapid accumulation stage. In addition, the expression level of *SmACCT* was higher than that of *SmDXR* at 70 and 100 DAF, suggesting that carbohydrate-derived carbon may be preferentially directed toward the MVA pathway for triterpenoid precursor formation in the pulp. Based on their pathway positions and expression patterns, *SmACCT*, *SmMVK*, and *SmIDI* were prioritized as candidate genes involved in precursor supply for saponin biosynthesis.

In the triterpenoid skeleton formation module, DEGs encoding *SmFPS*, *SmSS*, *SmSQE*, and *SmLUP2* showed relatively high expression during the early-to-middle developmental stages (70~100 DAF), followed by reduced expression at 130 DAF. These genes are involved in the conversion of IPP/DMAPP-derived intermediates toward FPP, squalene, 2,3-oxidosqualene, and amyrin-type triterpenoid skeletons. Their expression trends were broadly consistent with the major accumulation period of pulp saponins, indicating that triterpenoid skeleton formation was transcriptionally active during the early and middle stages of pulp development.

**Figure 5:**
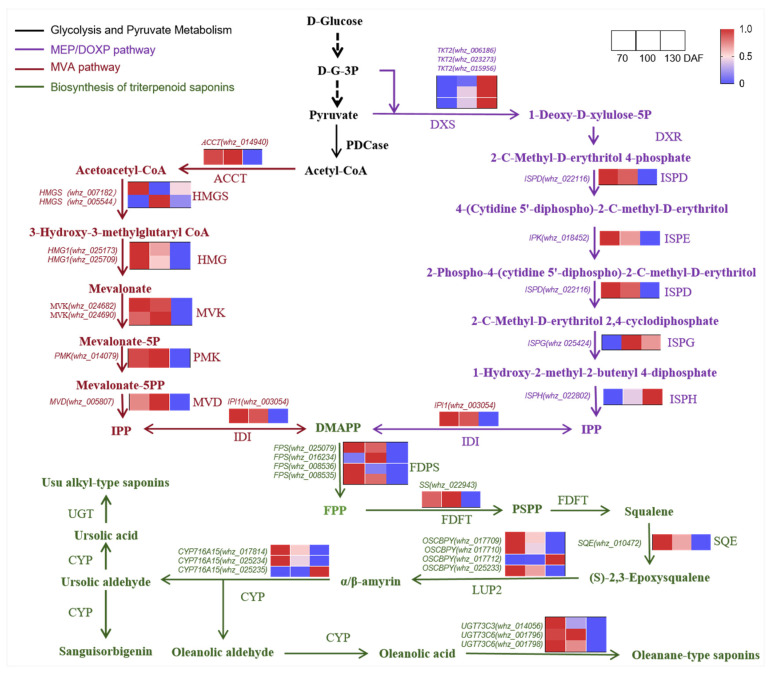
Expression pattern of triterpenoid saponin biosynthesis genes in pulp of *S. Saponaria*. Note: ACCT: acetyl-CoA C-acetyltransferase, MVK: mevalonate kinase, HMGS: hydroxymethylglutaryl-CoA synthase, HMG: hydroxymethylglutaryl-CoA reductase (NADPH), PMK: phosphomevalonate kinase, MVD: diphosphomevalonate decarboxylase; DXS: 1-deoxy-D-xylulose-5-phosphate synthase, DXR: 1-deoxy-D-xylulose-5-phosphate reductoisomerase, ISPD: 2-C-methyl-D-erythritol 4-phosphate cytidylyltransferase, ISPE: 4-diphosphocytidyl-2-C-methyl-D-erythritol kinase, ISPG: (E)-4-hydroxy-3-methylbut-2-enyl-diphosphate synthase, ISPH: 4-hydroxy-3-methylbut-2-en-1-yl diphosphate reductase, IDI: isopentenyl-diphosphate delta-isomerase, FPP: farnesyl pyrophosphate, DMAPP: dimethylallyl-PP, IPP: isopentenyl-PP, GPP: geranyl-PP, PSPP: presqualene diphosphate, FDPS: farnesyl diphosphate synthase, FDFT: farnesyl-diphosphate farnesyltransferase, SQLE: squalene monooxygenase, LUP2: lupeol synthase 2, CYP: cytochrome P450, UGT: UDP-glycosyltransferase.

For downstream modification, three DEGs encoding cytochrome P450 monooxygenases (*SmCYP716A15*) and three DEGs encoding UDP-glycosyltransferases (*SmUGT73C3*) were identified. These genes also displayed relatively high expression at 70 and 100 DAF, suggesting that oxidation and glycosylation reactions may contribute to the formation of diverse oleanane-type saponins in the pulp. Taken together, these results indicate that saponin biosynthesis in the pulp of *S. mukorossi* may mainly depend on the MVA pathway, and that *SmACCT*, *SmMVK*, *SmIDI*, *SmFPS*, *SmSm*, *SmSQE*, *SmLUP2*, *SmCYP716A15*, and *SmUGT73C3* represent the major candidate genes associated with pulp triterpenoid saponin accumulation (Figure 5).

### 2.9. Regulation of Sugar Metabolism and Carbon Allocation Mechanism in S. mukorossi Kernels and Pulp

#### 2.9.1. Sugar Metabolism Provides Energy and Precursors for Oil and Saponin Biosynthesis

To clarify how carbohydrate metabolism contributes to oil and saponin biosynthesis, DEGs involved in sucrose metabolism, glycolysis, and pyruvate metabolism were analyzed in the kernels and pulp of *S. mukorossi*. These pathways provide soluble sugars, carbon skeletons, energy, and acetyl-CoA, which are required for both fatty acid biosynthesis and triterpenoid saponin biosynthesis.

In the sucrose metabolism pathway, DEGs encoding *SmINV*, *SmSUS*, and *SmHXK* were detected in both kernels and pulp. These genes were generally highly expressed during the early-to-middle developmental stages (70~100 DAF), indicating active sucrose cleavage and hexose phosphorylation during the early and middle stages of fruit development. In the kernels, the relatively high expression of these genes may provide hexose-derived substrates for glycolysis and further acetyl-CoA formation, thereby supporting fatty acid and oil biosynthesis. In the pulp, sucrose metabolism may not only provide carbon skeletons through glycolysis but also supply UDP-glucose, which can serve as a glycosyl donor for saponin modification.

**Figure 6:**
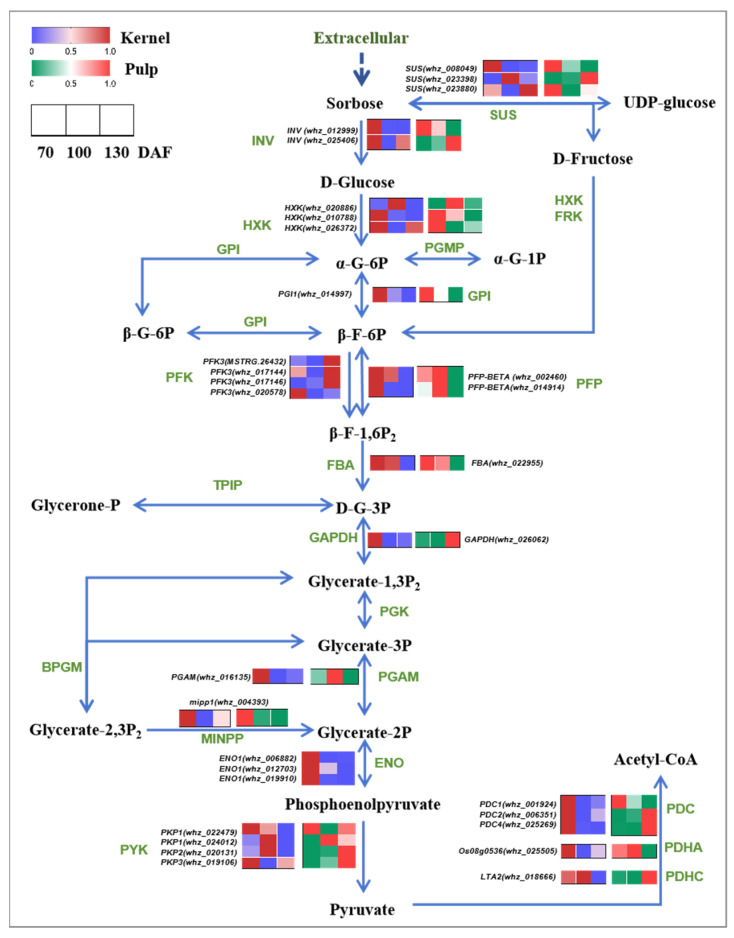
Expression patterns of genes involved in glycolysis and pyruvate metabolism in *S. mukorossi* kernels and pulp. Note: INV: sucrose invertase, HXK: hexokinase, SUS: sucrose synthase, GPI: glucose-6-phosphate isomerase, GAPDH: glyceraldehyde 3-phosphate dehydrogenase, PFP: diphosphate-dependent phosphofructokinase, PFK: 6-phosphofructokinase, PGK: phosphoglycerate kinase, PGAM: 2,3-bisphosphoglycerate-dependent phosphoglycerate mutase, ENO: enolase, PYK: pyruvate kinase, PDC: pyruvate dehydrogenase, PDHA: pyruvate dehydrogenase E1 component, PDHC: pyruvate dehydrogenase E2 component, D-G-3P: D-glyceraldehyde-3-phosphate, Glycerone-P: dihydroxyacetone phosphate, Glycerate-1,3P_2_: D-glycerate-1,3-bisphosphate, Glycerate-3P: 3-phospho-D-glycerate, Glycerate-2P: 2-phospho-D-glycerate, Glycerate-2,3P_2_: D-glycerate-2,3-bisphosphate, α-G-6P: α-D-glucose-6-phosphate, β-G-6P: β-D-glucose-6-phosphate, β-F-6P: β-D-fructose-6-phosphate, β-F-1,6P_2_: β-D-fructose-1,6-bisphosphate.

In the glycolysis and pyruvate metabolism pathways, several DEGs, including *SmGPI*, *SmGAPDH*, *SmENO*, *SmPYK*, and *SmPDC*, showed stage- and tissue-dependent expression patterns. In general, genes associated with glycolytic flux and pyruvate-to-acetyl-CoA conversion exhibited relatively high expression during the early developmental stage, especially in the kernels. This suggests that carbohydrate metabolism was more active during early kernel development and may contribute to the supply of acetyl-CoA for subsequent oil accumulation. In the pulp, these pathways may provide both acetyl-CoA for the MVA pathway and glycosyl precursors for triterpenoid saponin biosynthesis. Taken together, these results indicate that sugar metabolism provides the metabolic basis for the tissue-specific synthesis of oil in kernels and saponins in pulp (Figure 6).

#### 2.9.2. Tissue-Specific Carbon Allocation Toward Kernel Oil and Pulp Saponin Biosynthesis

To further evaluate the effect of sugar metabolism on oil and saponin biosynthesis, the expression patterns of genes involved in carbohydrate metabolism, fatty acid and oil biosynthesis, and triterpenoid saponin biosynthesis were integrated and compared between kernels and pulp. The results showed clear tissue-specific expression patterns corresponding to the differential accumulation of oil and saponins.

**Figure 7:**
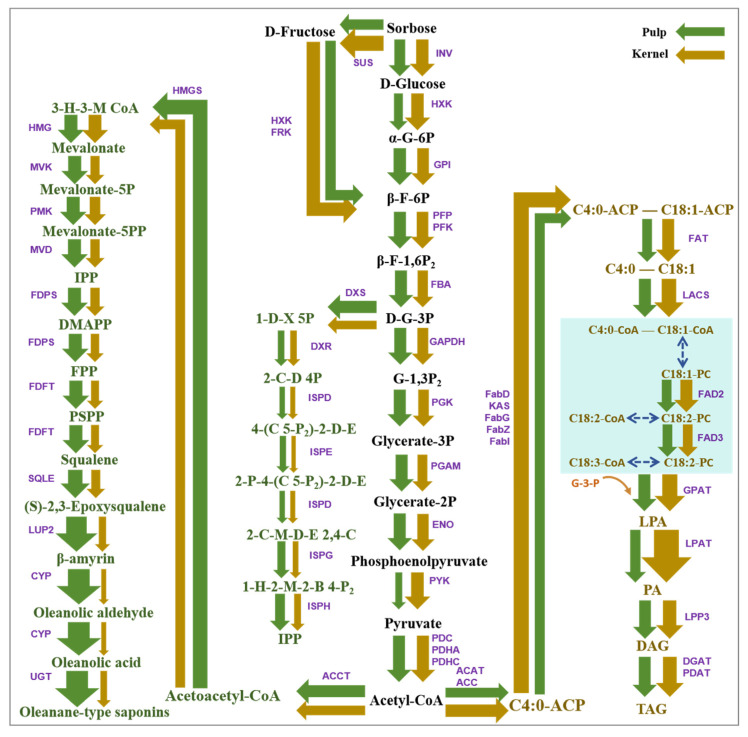
The influence of sugar metabolism on the synthesis of kernel oil and pulp saponins in *S. mukorossi.* Note: The thickness of the arrow represents the average difference in FPKM values of enzymes corresponding to the kernel and pulp between S1 and S3. ACAT: Acetyl-CoA: ACP S-acetyltransferase, ACC: acetyl-CoA carboxylase, Fab: DACP-S-malonyltransferase, KAS: 3-oxoacyl-ACP synthase, FabG: 3-oxoacyl-ACP reductase, FabZ: 3-hydroxyacyl-ACP dehydratase, FabI: enoyl-ACP reductase I, FAT: fatty acyl-ACP thioesterase, LACS: long-chain acyl-CoA synthetase, FAD2: ω-6 fatty acid desaturase, FAD3: ω-3 fatty acid desaturase, GPAT: glycerol-3-phosphate acyltransferase, LPAT: lysocardiolipin and lysophospholipid acyltransferase, LPP3: diacylglycerol diphosphate phosphatase, DGAT: diacylglycerol O-acyltransferase, PDAT: phospholipid: diacylglycerol acyltransferase, ACCT: acetyl-CoA C-acetyltransferase, HMGS: hydroxymethylglutaryl-CoA synthase, HMG: hydroxymethylglutaryl-CoA reductase (NADPH), MVK: mevalonate kinase, PMK: phosphomevalonate kinase, FDPS: farnesyl diphosphate synthase, FDFT: Farnesyl-diphosphate farnesyltransferase, SQLE: squalene monooxygenase, LUP2: lupeol synthase 2, CYP: Cytochrome P450, UGT: UDP-glycosyltransferase, DXS: 1-deoxy-D-xylulose-5-phosphate synthase, DXR: 1-deoxy-D-xylulose-5-phosphate reductoisomerase, ISPD: 2-C-methyl-D-erythritol 4-phosphate cytidylyltransferase, ISPE: 4-diphosphocytidyl-2-C-methyl-D-erythritol kinase, ISPG: (E)-4-hydroxy-3-methylbut-2-enyl-diphosphate synthase, ISPH: 4-hydroxy-3-methylbut-2-en-1-yl diphosphate reductase, LPA: lysophosphatidic acids, PA: Phosphatidic acid, DAG: diacylglycerol, TAG: triacylglycerol, PC: phosphatidylcholine, 3-H-3-M CoA: 3-Hydroxy-3-methylglutaryl CoA, 1-D-X 5P: 1-Deoxy-D-xylulose-5P, 2-C-D 4P: 2-C-Methyl-D-erythritol 4-phosphate, 2-P-4-(C 5P_2_)-2-D-E: 2-Phospho-4-(cytidine 5′-diphospho)-2-C-methyl-D-erythritol, 4-(C 5-P_2_)-2-D-E: 4-(Cytidine 5′-diphospho)-2-C-methyl-D-erythritol, 2-C-M-D-E 2,4-C: 2-C-Methyl-D-erythritol 2,4-cyclodiphosphate, 1-H-2-M-2-B 4P_2_: 1-Hydroxy-2-methyl-2-butenyl 4-diphosphate, DMAPP: dimethylallyl-PP, IPP: isopentenyl-PP, FPP: farnesyl-PP, PSPP: presqualene diphosphate.

In the kernels, several genes involved in sucrose metabolism and glycolysis, such as *SmINV*, *SmSUS*, *SmHXK*, *SmPGAM*, and *SmPYK*, showed relatively higher expression than in the pulp. Meanwhile, genes associated with fatty acid and oil biosynthesis, including *SmACC*, *SmLACS*, *SmGPAT*, and *SmLPAT*, were preferentially expressed in the kernels. These expression patterns were consistent with the high oil content observed in the kernels, suggesting that carbohydrate-derived carbon skeletons were mainly directed toward fatty acid and oil biosynthesis in this tissue. By contrast, in the pulp, genes involved in the MVA pathway and downstream triterpenoid saponin biosynthesis showed relatively higher expression than oil biosynthesis-related genes. In particular, *SmACCT*, *SmSQE*, *SmLUP2*, *SmCYP*, and *SmUGT* displayed relatively high expression in the pulp, which was consistent with the accumulation pattern of pulp saponins. In addition, the expression levels of genes in the MVA pathway were generally higher than those in the MEP/DOXP pathway, further supporting the idea that pulp saponin biosynthesis may mainly depend on the MVA pathway. Overall, these results support a tissue-specific carbon allocation model in *S. mukorossi* fruit: in the kernels, carbohydrate-derived carbon is preferentially directed toward fatty acid and oil biosynthesis, whereas in the pulp, carbon is preferentially allocated to the MVA-dependent triterpenoid saponin biosynthesis pathway. This differential allocation provides a transcriptional explanation for the high oil content in kernels and the high saponin content in pulp (Figure 7).

### 2.10. Potential Sugar-Hormone Regulatory Networks Associated with Oil and Saponins Biosynthesis in S. mukorossi

**Figure 8:**
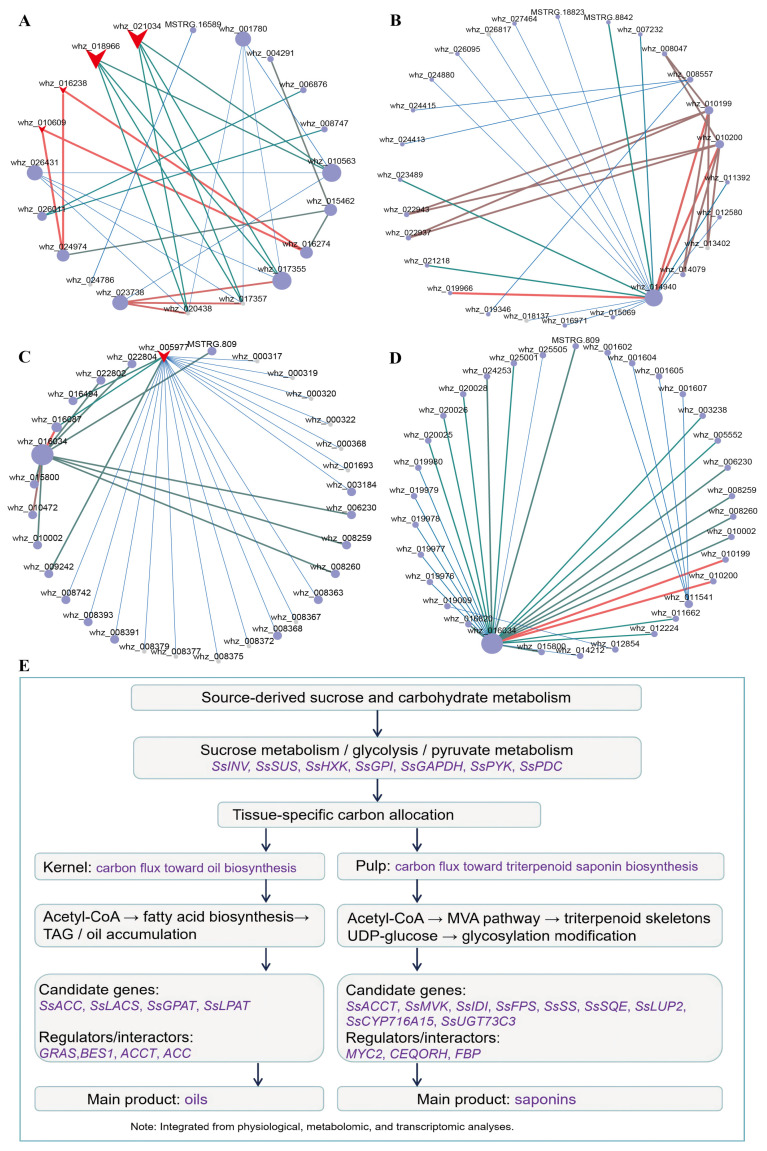
Transcription factor target and protein–protein interaction networks associated with kernel oil and pulp saponin biosynthesis in *S. mukorossi*. Note: (**A**): Analysis of transcription factor targeting for kernel oil biosynthesis. (**B**): Analysis of the protein–protein interaction network for kernel oil synthesis. (**C**): Analysis of transcription factor targeting for pulp saponin biosynthesis. (**D**): Analysis of the protein–protein interaction network for pulp saponin biosynthesis. (**E**): Proposed model showing tissue-specific carbon allocation toward oil biosynthesis in kernels and triterpenoid saponin biosynthesis in pulp.

To further explore the potential connection between carbohydrate metabolism and hormone signaling during tissue-specific oil and saponin accumulation, DEGs involved in hormone signal transduction, sugar metabolism, oil biosynthesis, and saponin biosynthesis were integrated for transcription factor target prediction and protein–protein interaction network analysis. These analyses were performed to identify candidate regulatory factors and interacting proteins that may connect sugar metabolism with kernel oil biosynthesis or pulp saponin biosynthesis. Figure 8 summarizes the predicted transcription factor target networks, key protein–protein interaction nodes, and the proposed model of tissue-specific carbon allocation in *S. mukorossi* fruit.

#### 2.10.1. Potential of Sugar-Hormone Regulatory Network Associated with Kernel Oil Biosynthesis

Statistical analysis of metabolic differentials, including hormones and sugars, in *S*. *mukorossi* kernels during the slow oil accumulation period (70 DAF) and the rapid oil accumulation period (100 DAF) revealed decreases in sucrose and glucose content, an increase in ZT and JA content, and decreases in IAA, ABA, and GA3 content. These changes suggest that sugar utilization and hormone signaling may be associated with the transition from slow to rapid oil accumulation in kernels.

Two DEG transcription factors, BES1, were annotated in the glycolytic pathway (ko00010), starch and sucrose metabolism pathway (ko00500), and pyruvate metabolism pathway (ko00620) of the kernels. In the plant hormone signal transduction pathway (ko04075) of the kernels, 33 DEGs were annotated, encompassing 23 enzyme-encoding genes and 8 types of transcription factors.

Transcription factor target analysis of the DEGs involved in hormone signal transduction, sugar metabolism, and oil biosynthesis identified *whz_021034 (GRAS)*, *whz_018966 (GRAS)*, *whz_016238 (BES1)*, and *whz_010609 (BES1)* as important candidate regulatory factors associated with kernel oil biosynthesis. GRAS and BES1 are transcription factors related to gibberellin and brassinosteroid signaling pathways, respectively. These results indicate that hormone-related transcription factors may participate in coordinating sugar metabolism and oil biosynthesis in kernels (Figure 8A).

Protein–protein interaction network analysis of the DEGs from the hormone signal transduction, sugar metabolism, and oil synthesis pathways identified *whz_014940 (ACCT)*, *whz_010200 (ACC)*, and *whz_010199 (ACC)* as key interacting proteins linking sugar metabolism and oil synthesis in the kernels. These proteins are associated with acetyl-CoA-related metabolism and fatty acid biosynthesis, suggesting that carbohydrate-derived carbon may be further directed toward kernel oil accumulation through these network nodes (Figure 8B).

#### 2.10.2. Potential Sugar-Hormone Regulatory Network Associated with Pulp Saponin Biosynthesis

Statistical analysis of differential metabolites, including hormones and sugars, in *S. mukorossi* pulp during the rapid saponin accumulation stage at 70 DAF and the slow saponin accumulation stage at 100 DAF showed that five metabolites were annotated in the plant hormone signal transduction pathway (ko04075). Among them, jasmonoyl-L-isoleucine, IAA, glucose, and sucrose were up-regulated, whereas salicylic acid was down-regulated. Meanwhile, 174 DEGs were annotated in this pathway, including 57 enzyme-encoding genes. These results suggest that sugar metabolism and hormone signaling, especially jasmonic acid-related signaling, may be associated with pulp saponin accumulation.

Transcription factor target analysis of DEGs involved in hormone signal transduction, sugar metabolism, and saponin biosynthesis identified *whz_005977 (MYC2)* as an important candidate regulatory factor associated with pulp saponin biosynthesis. MYC2 is a key transcription factor in the jasmonic acid signaling pathway and may participate in linking hormone signaling with triterpenoid saponin biosynthesis in the pulp (Figure 8C).

Protein–protein interaction network analysis of DEGs involved in hormone signal transduction, sugar metabolism, and saponin biosynthesis identified *whz_016034 (CEQORH)* and *whz_011541 (FBP)* as key interacting proteins linking sugar metabolism and saponin biosynthesis in the pulp. The close interactions among *whz_016034 (CEQORH)*, *whz_010200 (ACC)*, *whz_010199 (ACC)*, and *MSTRG.809 (ALDH)* further suggest a potential connection between carbon metabolism and downstream metabolic conversion during pulp saponin biosynthesis (Figure 8D).

Based on the above transcription factor target analysis, protein interaction network analysis, and integrated physiological and transcriptomic results, a proposed model was constructed to summarize the tissue-specific carbon allocation and regulatory network in *S. mukorossi* fruit (Figure 8E). It should be noted that oil and saponin contents represent cumulative metabolite pools, whereas gene expression reflects stage-specific transcriptional activity. Therefore, candidate genes were mainly evaluated based on their pathway positions, tissue-specific expression patterns, and consistency with the active accumulation phases of oil and saponins.

### 2.11. qRT-PCR Validation

**Figure 9:**
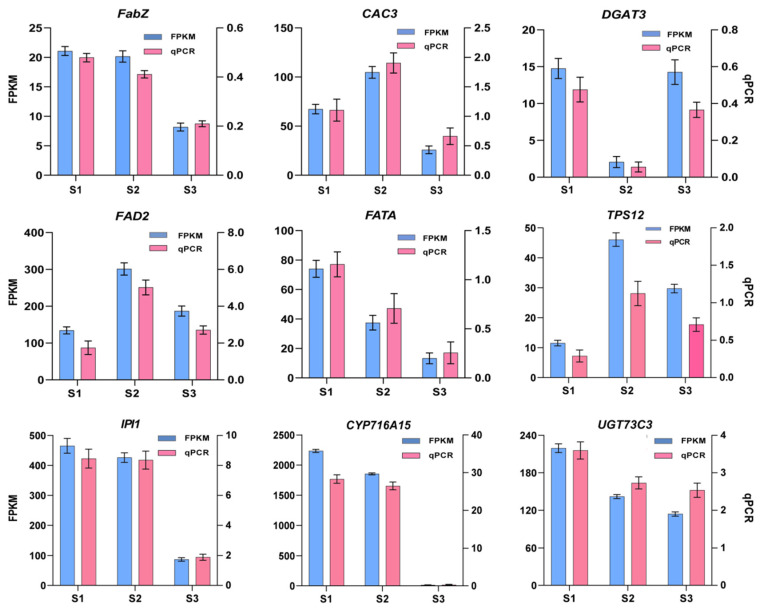
qRT-PCR validation of the transcriptome in *S. mukorossi* kernels and pulp.

To verify the reliability of the RNA-seq data and to support the key transcriptomic findings related to tissue-specific oil and saponin biosynthesis, nine representative DEGs were selected for qRT-PCR validation. These genes were chosen based on their pathway functions, tissue-specific expression patterns, and relevance to the main conclusions of this study. Five genes involved in fatty acid and oil biosynthesis in kernels, including *SmfabZ*, *SmCAC3*, *SmFATA*, *SmFAD2*, and *SmDGAT3*, were selected to represent fatty acid synthesis, fatty acid desaturation, and triacylglycerol assembly. Four genes involved in triterpenoid saponin biosynthesis in pulp, including *SmTPS12*, *SmIPI1*, *SmCYP716A15*, and *SmUGT73C3*, were selected to represent precursor conversion, triterpenoid skeleton formation, oxidation, and glycosylation modification. The qRT-PCR results showed expression trends consistent with the RNA-seq data, confirming the reliability of the transcriptome analysis and supporting the identification of candidate genes associated with kernel oil accumulation and pulp saponin accumulation (Figure 9).

## 3. Discussion

### 3.1. Fruit Development and the Accumulation Patterns of Oil and Saponins in S. mukorossi

In this study, the fruit development process of *S. mukorossi* was systematically characterized. Flower buds developed in late April, the initial fruit stage appeared in mid-June, and fruit maturity was reached at approximately 130 DAF. The developmental process could be divided into four stages: slow growth, rapid expansion, color transition, and maturation, which is generally consistent with previous observations reported by Xu Y.Y. et al. [12] and Zhou X. et al. [12]. During early development, the kernel remained in a liquid embryo state from 0 to 50 DAF, and a solid kernel began to form at approximately 60 DAF, indicating the beginning of kernel differentiation and subsequent storage metabolism.

The accumulation patterns of oil and saponins showed clear tissue specificity. Kernel oil content followed a typical S-shaped pattern, with a slow accumulation phase, a rapid accumulation phase, and a stabilization phase. In contrast, pulp saponin content increased continuously before maturity and then tended to stabilize. These results indicate that kernel oil and pulp saponins are the two major tissue-specific products of *S. mukorossi* fruit. The different accumulation patterns also suggest that the kernel and pulp act as distinct sink tissues during fruit development, with carbon being preferentially allocated to storage oil in the kernel and to triterpenoid saponins in the pulp.

### 3.2. Metabolic Features of the Kernel and Pulp

Fatty acid analysis showed that the mature kernels of *S. mukorossi* contained abundant unsaturated fatty acids, with C18:1 and C18:3 being the dominant components. This composition is similar to that reported in *Styrax tonkinensis* [13], but differs from that of *Xanthoceras sorbifolium* [14], *Swida wilsoniana* [15], and *Symplocos paniculata* [16], in which C18:1 and C18:2 are the major fatty acids. The high proportion of unsaturated fatty acids indicates that *S. mukorossi* kernel oil has potential value for oleochemical and bioenergy applications.

In the pulp, LC-MS/MS analysis revealed abundant secondary metabolites, including flavonoids, phenolic acids, and terpenoids. These compounds may be associated with the antioxidant, anti-inflammatory, and antimicrobial properties of *S. mukorossi* pulp [17]. KEGG enrichment analysis further showed that differential metabolites were mainly enriched in pathways related to secondary metabolite biosynthesis, ABC transporters, and plant secondary metabolite biosynthesis. These findings suggest that the pulp maintains active secondary metabolism during development, which may contribute not only to saponin accumulation but also to fruit defense and maturation processes [18].

### 3.3. Transcriptomic Evidence for Tissue-Specific Metabolic Priorities

Transcriptome analysis revealed distinct gene expression patterns between kernels and pulp during fruit development. The number of DEGs in the pulp was higher than that in the kernels, suggesting that pulp development involves more extensive transcriptional regulation. Both tissues showed relatively high transcriptional activity at the early developmental stage, followed by an overall decrease in gene expression from 70 to 130 DAF. This pattern is consistent with the idea that many developmental and metabolic regulatory processes are initiated during early fruit development and then gradually shift toward metabolite accumulation and maturation.

KEGG enrichment analysis showed that metabolic pathways were highly represented in both tissues. In kernels, DEGs were mainly associated with lipid metabolism, whereas in pulp, DEGs were preferentially enriched in terpenoid and polyketide metabolism. In addition, carbohydrate metabolism and secondary metabolite biosynthesis were enriched in both tissues. These results support the hypothesis that carbohydrate metabolism provides a shared carbon source, while downstream carbon allocation differs between kernels and pulp according to their tissue-specific metabolic priorities.

For pulp saponin biosynthesis, both the MVA and MEP/DOXP pathways can provide the isoprenoid precursors IPP and DMAPP. The MVA pathway uses acetyl-CoA as the initial substrate, whereas the MEP/DOXP pathway starts from pyruvate and glyceraldehyde-3-phosphate [19]. Previous studies have suggested that the MVA pathway plays an important role in triterpenoid saponin biosynthesis in *S. mukorossi*, and that UGT genes participate in glycosylation modification [20]. In this study, the MVA pathway-related genes *SmACCT*, *SmMVK*, and *SmIDI* showed relatively high expression at 70 and 100 DAF, whereas several MEP/DOXP pathway genes showed lower expression. This suggests that precursor supply for pulp saponin biosynthesis may mainly depend on the MVA pathway. In addition, *SmFPS*, *SmSS*, *SmSQE*, and *SmLUP2* may participate in triterpenoid skeleton formation, while *SmCYP716A15* and *SmUGT73C3* may be involved in downstream oxidation and glycosylation reactions. CYPs and UGTs are widely recognized as key enzyme families responsible for the structural diversification of triterpenoid saponins [21,22].

### 3.4. Sugar Metabolism and Differential Carbon Allocation Between Oil and Saponin Biosynthesis

Carbohydrate metabolism is central to fruit development because it provides energy, carbon skeletons, and metabolic precursors for both storage and specialized metabolism. Sucrose transported from source leaves is unloaded into fruits and further cleaved by enzymes such as INV to provide glucose and fructose for downstream metabolism [23]. In this study, genes involved in sucrose metabolism, glycolysis, and pyruvate metabolism, including *SmINV*, *SmSUS*, *SmHXK*, *SmGPI*, *SmGAPDH*, *SmPDC*, *SmPDHA*, and *SmPDHC*, were identified in kernels and pulp, suggesting that sugar metabolism provides the metabolic basis for both kernel oil and pulp saponin biosynthesis.

The expression patterns of sugar metabolism-related genes differed between the two tissues. In kernels, several genes involved in sucrose cleavage, glycolysis, and pyruvate-to-acetyl-CoA conversion showed relatively high expression, indicating active carbon conversion during kernel development. This may provide acetyl-CoA and energy for fatty acid and triacylglycerol biosynthesis. In agreement with this, oil biosynthesis-related genes, including *SmACAT*, *SmACC*, *SmLACS*, *SmGPAT*, and *SmLPAT*, were preferentially expressed in kernels. By contrast, in pulp, genes associated with the MVA-dependent triterpenoid saponin pathway and downstream modification were more closely associated with saponin accumulation. These results indicate that carbohydrate-derived carbon is differentially allocated in the two tissues: toward fatty acid and oil biosynthesis in kernels and toward triterpenoid saponin biosynthesis in pulp.

### 3.5. Sugar–Hormone Regulatory Networks and Practical Implications

The integration of sugar metabolites, hormone-related metabolites, DEGs, transcription factor target prediction, and protein–protein interaction analysis further suggested that sugar metabolism may interact with hormone signaling to regulate tissue-specific carbon allocation. In kernels, the transition from slow to rapid oil accumulation was accompanied by decreases in sucrose and glucose and increases in ZT and JA. GRAS and BES1 transcription factors, which are related to GA and BR signaling, respectively, may participate in coordinating hormone signaling, sugar metabolism, and oil biosynthesis [24,25,26,27]. Protein interaction analysis further identified ACCT and ACC as key network nodes associated with acetyl-CoA-related metabolism and fatty acid biosynthesis. In pulp, MYC2, a transcription factor associated with jasmonic acid signaling, was identified as a potential regulatory node linking hormone signaling and triterpenoid saponin biosynthesis. CEQORH and FBP were also identified as interacting proteins that may connect carbon metabolism with saponin biosynthesis. Although these regulatory relationships require further functional validation, they provide useful clues for understanding how sugar metabolism, hormone signaling, and tissue-specific biosynthetic pathways are coordinated during *S. mukorossi* fruit development.

These results also have implications for the breeding and cultivation of *S. mukorossi*. The different accumulation patterns of kernel oil and pulp saponins suggest that high-oil kernels and high-saponin pulp can be considered as two related but separate selection targets. In future genotype evaluation, materials with high kernel oil content, high pulp saponin content, or a favorable balance between the two traits could be prioritized. The candidate genes identified in lipid biosynthesis, MVA-dependent triterpenoid saponin biosynthesis, and sugar metabolism may also be useful for subsequent functional validation and marker development. In addition, the close relationship between carbohydrate metabolism and oil or saponin accumulation suggests that cultivation practices affecting carbon assimilation, sucrose transport, and source–sink balance may influence the production of these two valuable products. Therefore, this study provides a physiological and molecular basis for improving the production and utilization of *S. mukorossi* fruit.

## 4. Materials and Methods

### 4.1. Materials

Three healthy 8-year-old *S. mukorossi* trees at the full-bearing stage were selected from a *S. mukorossi* germplasm resource nursery in Weixin Town, Shimen County, Hunan Province, China (29.766501 N, 111.127309 E). The trees were grown under similar field management conditions and showed normal growth and fruiting, without visible disease, insect damage, or mechanical injury. The selected trees had an average height of approximately 6.5 m, an average diameter at breast height of 22.5 cm, and an average crown spread of approximately 5.0 m × 6.0 m. Trees with similar age, growth vigor, trunk diameter, and canopy size were used to minimize variation among individual plants.

Fruit sampling was conducted from June to October 2023. Fruits were collected every 10 days from 10 to 160 days after flowering (DAF) to follow changes in fruit development, oil and saponin accumulation, and carbohydrate metabolism. At each sampling stage, fruits were collected from the outer upper canopy branches on the eastern, southern, western, and northern sides of each tree. Fruits with similar developmental status and no visible disease, insect damage, or mechanical injury were selected. A total of 45 fruits were collected at each stage, with three biological replicates. Each replicate consisted of 15 fruits collected from one tree. Sampling was carried out on rain-free mornings between 09:00 and 11:00.

After collection, part of the fruits was dissected using pruning shears. The kernels and pulp (exocarp and mesocarp) were separated, wrapped in tin foil, immediately frozen in liquid nitrogen, transported to the laboratory, and stored at −80 °C for subsequent transcriptome sequencing, qRT-PCR validation and endogenous hormone analysis. The remaining portion was used for the determination of oil, saponin, endogenous hormones and sugar contents.

### 4.2. Fruit Morphological

Use D7100 camera (Nikon Corporation, Tokyo, Japan) to take photos of *S. mukorossi* fruit, and use one ten thousandth electronic analytical balance FA2004B (Shanghai Yueping Instrument Co., Ltd., Shanghai, China) to measure the fresh weight of 100 fruits (FWF), fresh weight per fruit (FWPF) and dry weight per fruit (DWPF), repeating five times. Measure the fruit longitudinal diameter (FLD), maximum transverse diameter (FDL), minimum transverse diameter (FDW), seed longitudinal diameter (SLD), maximum transverse diameter (SDL), minimum transverse diameter (SDW), and pulp thickness (PT) using a vernier caliper, and repeat five times.

### 4.3. Oil Extraction and Relative Composition of Fatty Acids

The oil content of *S. mukorossi* kernels and pulp were determined from dried powder samples by Soxhlet extraction, following the Chinese national standard “Determination of Fat in Foods” (GB 5009.6-2016) [28]. Methods for sample pretreatment and oil extraction were based on Zhang L.H. et al. [15] and Zhou X. et al. [2], respectively. An SZE-101 fat analyzer (Shanghai ShineJan Instruments, Shanghai, China) was employed to quantify the oil content of the kernels and pulp. Specifically, about 4.0 g of dried powder (M_0_) was extracted with petroleum ether (99.7%, 30~60 °C) for over 6 h. The residues were then dried at 105 °C for 2 h and weighed (M_1_). Oil content (W) was calculated as: W = (M_0_ − M_1_)/M_0_ × 100%. Weight measurements were accurate to 0.0001 g. The process was repeated three times.

The determination of fatty acid methyl esters and their relative content follows the Chinese national standard “Determination of Fat in Foods” (GB 5009.6-2016) [28]. Gas chromatography (Shimadzu Nexis GC-2030, Kyoto, Japan) is used to determine the relative content of fatty acids in kernels and pulp. The specific operating steps and GC program settings for fatty acid methyl esters refer to the method of Zhou X. et al. [2].

### 4.4. Extraction and Estimation of Total Saponin Content

The extraction and determination of total saponins were performed with reference to the methods described by Xu Y.Y. et al. [12] and Chen C.Y. et al. [29], with minor modifications. Dried powder samples of *S. mukorossi* kernels and pulp were used for saponin extraction and estimation. Briefly, 5.0 g of dried sample powder was extracted with water at a solid-to-liquid ratio of 1:10 (*w*/*v*). The extraction pH was adjusted to 5.0, and a compound enzyme preparation consisting of pectinase and cellulase at a ratio of 2:1 was added at 7.0% of the dry sample weight. The mixture was shaken at 55 °C for 2 h and then centrifuged at 5000 rpm for 30 min. The supernatant was collected and freeze-dried. The extraction procedure was performed in triplicate.

The total saponin content was estimated by high-performance liquid chromatography based on UV absorbance signals monitored at 210 nm, using oleanolic acid and hederagenin as reference standards. The freeze-dried extract was redissolved in methanol, filtered through a 0.22 μm membrane filter, and subjected to HPLC analysis. The mobile phase consisted of water containing 0.4% H_3_PO_4_ and methanol at a ratio of 16:84 (*v*/*v*). The column temperature was 30 °C, the flow rate was 1.0 mL/min, and the injection volume was 10 μL.

Standard solutions of oleanolic acid and hederagenin were prepared at the following concentration combinations: 1 and 5 mg/L, 4 and 20 mg/L, 10 and 50 mg/L, 20 and 100 mg/L, 50 and 250 mg/L, 80 and 400 mg/L, and 100 and 500 mg/L, respectively. Calibration curves were established by plotting peak area (Y) against standard concentration (X, mg/L). The linear regression equations were Y = 12,625X − 6633.6 (R^2^ = 0.9991) for oleanolic acid and Y = 9434.4X + 9392.6 (R^2^ = 0.9995) for hederagenin.

The contents of oleanolic acid equivalents and hederagenin equivalents in the samples were calculated using the corresponding external calibration curves. The estimated total saponin content was expressed as the sum of oleanolic acid and hederagenin equivalents on a dry weight basis. This assay was mainly used to compare the relative changes in total saponin accumulation between tissues and developmental stages, rather than to identify or accurately quantify individual saponin compounds.

### 4.5. Soluble Sugar Content Determination

The contents of glucose, fructose, and sucrose in the fruit were determined using high-performance liquid chromatography with a refractive index detector (HPLC-RID), following the current Chinese national standard method for monosaccharide determination (GB5009.8—2023) [30]. A 2.0 g sample of dried *S. mukorossi* kernel and pulp powder was placed in a beaker containing 50 mL of deionized water. Then, 2 mL of 0.5% zinc acetate solution and 2 mL of 0.5% potassium ferrocyanide solution were added and thoroughly mixed. The mixture was extracted in an ultrasonic cleaner at 50 °C for 30 min (200 W), followed by a 10 min incubation in a 60 °C water bath. It was then shaken for 5 min to ensure complete reaction. After cooling to room temperature, the solution was diluted to 100 mL with deionized water.

Prior to instrumental analysis, the diluted solution was processed to remove solid particulate impurities. It was centrifuged at 12,000 r/min for 5 min. The supernatant was collected and filtered through a 0.45 µm aqueous cellulose membrane filter. Subsequently, 1 mL of the filtrate was passed through a C18 solid-phase extraction (SPE) column, which had been pre-conditioned with 1 mL of methanol followed by 1 mL of deionized water. The effluent was collected, and the column was rinsed with 1 mL of deionized water. The rinse solution was combined with the initial effluent, and the total volume was adjusted to 10 mL.

A mixed standard stock solution of glucose, fructose, and sucrose was prepared at a concentration of 20 mg/mL each. Using this mixed stock solution as a baseline, a series of working standard solutions were prepared by dilution to concentrations of 0.2 mg/mL, 2 mg/mL, 4 mg/mL, 6 mg/mL, and 10 mg/mL (with 5 mL prepared for each concentration). An aliquot of 10 µL from each concentration level was injected for analysis to construct a calibration curve plotting peak area against concentration.

Chromatographic Conditions: Column: Comasil NH_2_ (Conomasil Instruments Technology Co., Ltd., Tianjin, China): 5 µm, 250 mm × 4.6 mm. Mobile phase: acetonitrile: water = 70:30 (*v*/*v*). Flow rate: 1.0 mL/min. Detector and column temperature: 40 °C. injection volume: 10.0 µL. Drift tube temperature: 80~90 °C. Nitrogen pressure: 350 kPa. Nitrogen flow rate: 2.5 L/min. Impacto: off. The standard curves were plotted with peak area on the Y-axis and the concentrations of glucose, fructose, and sucrose on the X-axis, respectively: Glucose: Y = 14,015.1X + 969.9 (R^2^ = 0.9995), Fructose: Y = 16,433.9X + 336.3 (R^2^ = 0.9996), Sucrose: Y = 17,391.6X + 456.6 (R^2^ = 0.9991).

The soluble starch content in the *S. mukorossi* kernel and pulp was determined using the anthrone-ethyl acetate colorimetric method. Six test tubes were taken, and 0.0 μg, 10.0 μg, 20.0 μg, 40.0 μg, 60.0 μg, and 80.0 μg of starch standard solution were added respectively. The volume in each tube was adjusted to 2.0 mL with distilled water. After cooling, 6.0 mL of anthrone-H_2_SO_4_ solution was added to each tube. The tubes were shaken thoroughly and cooled again. They were then placed in a boiling water bath for 5 min for color development, removed, and cooled once more. Using distilled water as a blank, the absorbance was measured at a wavelength of 620 nm. A standard curve was plotted with the Y-axis representing the absorbance value and the X-axis representing the starch concentration: Y = 0.0305x + 0.0078 (R^2^ = 0.9998). The significant linear relationship between X and Y confirms that this curve can be used as the standard curve for determining starch content in the kernel and pulp of *S. mukorossi*.

The soluble sugar content in the *S. mukorossi* kernel and pulp was determined using the anthrone colorimetric method. Seven test tubes were taken, and 0 mL, 0.1 mL, 0.2 mL, 0.3 mL, 0.4 mL, 0.5 mL, 0.6 mL, and 0.7 mL of a 100 μg/mL glucose standard solution were added to them, respectively. Then, 5.0 mL of anthrone reagent was added to each tube, and distilled water was added to bring the total volume to 6.0 mL. After thorough mixing, the tubes were placed in a boiling water bath for 10 min. Following cooling, the absorbance was measured at a wavelength of 620 nm. A standard curve was plotted with the Y-axis representing the absorbance value and the X-axis representing the glucose concentration: Y = 0.0515x − 0.001 (R^2^ = 0.9999). The significant linear relationship between X and Y indicates that this curve can be reliably used as the standard curve for determining soluble sugar content in the kernel and pulp of *S. mukorossi*.

### 4.6. Endogenous Hormone Content Determination

The contents of five endogenous hormones—Auxin (3-Indoleacetic acid, IAA), Zeatin (ZT), Abscisic acid (ABA), Gibberellin A3 (GA3), and Jasmonic acid (JA)—in *S. mukorossi* fruits at different developmental stages were determined using High-Performance Liquid Chromatography (HPLC) [26,27]. The specific procedures are as follows:

Preparation of standard solutions: precisely 0.05 g of each standard (IAA, ABA, GA3, ZT, and JA) was accurately weighed using a balance with 0.1 mg precision. Each standard was dissolved with chromatographic-grade methanol and diluted to 500 mL in a brown volumetric flask, preparing a mixed standard stock solution with a concentration of 100 mg/L for each component.

Sample Preparation: Precisely 1.0 g of the *S. mukorossi* kernel and pulp was weighed. Then, 10.0 mL of pre-cooled 80% methanol was added. The mixture was ground under light-protected conditions and poured into a 25 mL centrifuge tube. After extraction in the dark for 16 h, it was centrifuged at 6000 r/min for 10 min, and the supernatant was collected. The residue was subjected to two additional extraction cycles by adding approximately a 1:5 (*v*/*v*) ratio of 80% methanol, vortex-mixing, and centrifuging. The supernatants from all extractions were combined. Approximately 0.3 g of activated charcoal was added to the combined supernatant, which was then shaken for decolorization for 20 min before filtration. The filtrate was further passed through a C18 solid-phase extraction (SPE) column. The collected filtrate was evaporated to dryness under a stream of nitrogen. Subsequently, 10.0 mL of phosphate buffer (pH = 3.5) was added, and the mixture was shaken to dissolve. Liquid–liquid extraction was performed three times using an equal volume of ethyl acetate each time. The combined ethyl acetate phases were evaporated to dryness under nitrogen. The residue was reconstituted in 2.0 mL of chromatographic-grade methanol and filtered through a 0.22 μm organic membrane filter.

Chromatographic Conditions: column: Thermo U3000 syncronis C18 (250 mm × 4.6 mm, 5 μm, Thermo Fisher Scientific, Waltham, MA, USA). Column Temperature: 35 °C. Mobile phase: Phase A: Chromatographic-grade methanol, phase B: Aqueous acetic acid solution (pH = 3.2). Isocratic elution with V_(A)_:V_(B)_ = 4.5:5.5. Flow Rate: 1.0 mL/min. Injection volume: 20 μL. Calibration curve construction: The standard stock solution was diluted to prepare mixed standard solutions at concentrations of 0.25 mg/L, 0.5 mg/L, 2.5 mg/L, 12.5 mg/L, 25.0 mg/L, and 50.0 mg/L. These were filtered through a 0.22 μm organic membrane filter. Quantification was performed using the external standard method. HPLC analysis was conducted at specific detection wavelengths: 270 nm (for IAA, ZT, ABA), 230 nm (for JA), and 206 nm (for GA3). Calibration curves were plotted with peak area (Y-axis) against mass concentration (X-axis). The standard curves for each hormone were as follows: IAA: Y = 34,036 X + 2269 (R^2^ = 0.9999), ABA: Y = 80,356 X + 18,767 (R^2^ = 0.9997), GA3: Y = 21,566 X + 1331 (R^2^ = 0.999), ZT: Y = 70,214 X + 13,478 (R^2^ = 0.9998), JA: Y = 1288.8 X + 3599.1 (R^2^ = 0.9992).

### 4.7. Extraction and Estimation of Endogenous Hormone Contents

The contents of five endogenous hormones, including auxin/3-indoleacetic acid (IAA), zeatin (ZT), abscisic acid (ABA), gibberellin A3 (GA3), and jasmonic acid (JA), in *S. mukorossi* kernels and pulp at different developmental stages were estimated by high-performance liquid chromatography (HPLC) based on UV absorbance signals, according to previously reported methods with minor modifications [31,32].

For standard solution preparation, 0.05 g of each hormone standard, including IAA, ABA, GA3, ZT, and JA, was accurately weighed and dissolved in chromatographic-grade methanol. The solutions were diluted to 500 mL in amber volumetric flasks to prepare mixed standard stock solutions with a concentration of 100 mg/L for each component.

For sample extraction, 1.0 g of each *S. mukorossi* kernel or pulp sample was weighed and mixed with 10.0 mL of pre-cooled 80% methanol. The samples were ground under light-protected conditions and transferred into 25 mL centrifuge tubes. After extraction in the dark for 16 h, the mixtures were centrifuged at 6000 rpm for 10 min, and the supernatants were collected. The residues were extracted twice more with 80% methanol at a residue-to-solvent ratio of approximately 1:5 (*w*/*v*), followed by vortex mixing and centrifugation. The supernatants from the three extractions were combined.

Approximately 0.3 g of activated charcoal was added to the combined extract, and the mixture was shaken for 20 min for decolorization. After filtration, the filtrate was further purified using a C18 solid-phase extraction (SPE) column and then evaporated to dryness under a stream of nitrogen. Subsequently, 10.0 mL of phosphate buffer (pH 3.5) was added to dissolve the residue, and liquid–liquid extraction was performed three times with an equal volume of ethyl acetate. The combined ethyl acetate phases were evaporated to dryness under nitrogen. The residue was reconstituted in 2.0 mL of chromatographic-grade methanol and filtered through a 0.22 μm organic membrane filter before HPLC analysis.

Chromatographic separation was performed on a Syncronis C18 column (250 mm × 4.6 mm, 5 μm; Thermo Fisher Scientific, Waltham, MA, USA). The column temperature was maintained at 35 °C. The mobile phase consisted of chromatographic-grade methanol as phase A and aqueous acetic acid solution (pH 3.2) as phase B. Isocratic elution was performed with A:B = 4.5:5.5 (*v*/*v*) at a flow rate of 1.0 mL/min. The injection volume was 20 μL.

The mixed standard stock solution was diluted to prepare working standard solutions at concentrations of 0.25, 0.5, 2.5, 12.5, 25.0, and 50.0 mg/L. All standard solutions were filtered through a 0.22 μm organic membrane filter before analysis. Quantification was performed using the external standard method. UV absorbance signals were monitored at 270 nm for IAA, ZT, and ABA, 230 nm for JA, and 206 nm for GA3. Calibration curves were established by plotting peak area (Y) against mass concentration (X, mg/L). The regression equations were as follows: IAA, Y = 34,036X + 2269 (R^2^ = 0.9999); ABA, Y = 80,356X + 18,767 (R^2^ = 0.9997); GA3, Y = 21,566X + 1331 (R^2^ = 0.9990); ZT, Y = 70,214X + 13,478 (R^2^ = 0.9998); and JA, Y = 1288.8X + 3599.1 (R^2^ = 0.9992).

The hormone contents in the samples were calculated using the corresponding external calibration curves. Since isotope-labeled or compound-specific internal standards were not used in this assay, the hormone data were mainly used to compare relative changes among developmental stages under the same sample preparation and chromatographic conditions, rather than for high-precision absolute quantification.

### 4.8. Pulp Metabolome Determination

Pulp from *S. mukorossi* fruits at 70 DAF (GP-S1), 100 DAF (GP-S2), and 130 DAF (GP-S3) was selected for widely targeted metabolomic sequencing analysis. The metabolomic profiling of the pulp was conducted by Guangzhou Gidio Bio-Tech Co., Ltd. (Guangzhou, China). Metabolite extraction from the pulp was performed with reference to methods from Shi X.J. et al. [33] and Vos R.C.D. et al. [34], as follows: (1) Samples were freeze-dried and ground into powder. (2) An appropriate amount of powder was weighed into a centrifuge tube, and extraction solvent (volume ratio of methanol:water:powder = 3:1:1, containing internal standards) was added. (3) The mixture was vortexed for 30 s, homogenized at 40 Hz for 4 min, and subjected to ultrasound in an ice-water bath for 5 min. (4) Steps of homogenization and ultrasound were repeated three times. (5) The mixture was placed on a shaker at 4 °C for 12 h. (6) Centrifugation was performed at 4 °C and 12,000 rpm for 15 min. (7) The supernatant was collected, filtered through a 0.22 μm membrane filter, transferred to a 2 mL injection vial, and equal volumes from each sample were pooled to create a quality control (QC) sample. (8) Samples were stored at −80 °C until instrumental analysis. Analysis was performed using an ExionLC AD ultra-performance liquid chromatography system (SCIEX LLC, Framingham, MA, USA). Mobile phase A was 0.1% formic acid in water, and mobile phase B was acetonitrile. The column oven temperature was set at 40 °C, the autosampler temperature at 4 °C, and the injection volume was 2 μL.

### 4.9. RNA-Seq and Analysis

Kernels (ZR) and pulp (GP) of *S. mukorossi* fruits at three key developmental stages, 70 DAF (S1), 100 DAF (S2), and 130 DAF (S3), were selected for transcriptome sequencing analysis. The transcriptome sequencing of the kernels and pulp was conducted by Guangzhou Gidio Bio-Tech. Co., Ltd., with experimental procedures referencing the method described by Zhou X. et al. [2]. Raw sequencing data underwent quality control using fastp, and high-quality clean data were obtained after filtering out low-quality reads. De novo transcriptome assembly was performed using Trinity software (v2.15.1) [35]. The published *Sapindus mukorossi* whole genome served as the reference for alignment, and functional annotation was carried out by matching sequences against the KEGG public database. Gene expression levels for all genes were quantified using RSEM [36]. Differential gene expression analysis was performed using the DESeq2 software package [37], with a significance threshold set at an absolute fold change greater than 2. Genes with an FDR < 0.05 and |log2FC| > 1 (equivalent to log2(2)) were identified as differentially expressed.

### 4.10. qT-PCR Validation of RNA-Seq

To validate the RNA-seq results, nine representative DEGs associated with oil and saponin biosynthesis were selected for qRT-PCR analysis. Kernels were selected for five key genes involved in the lipid biosynthesis pathway: *SmfabZ*, *SmCAC3*, *SmFAD2*, *SmFATA*, and *SmDGAT3*, while pulp was selected for four key genes related to the saponin biosynthesis pathway: *SmTPS12*, *SmIPI1*, *SmUGT73C3*, and *SmCYP716A15*, based on their functional annotations, differential expression patterns, and relevance to the major metabolic pathways analyzed in this study (Table 2). *SmGAPCP1* (a housekeeping gene encoding glyceraldehyde-3-phosphate dehydrogenase) and *SmATHB-13* (a eukaryotic translation initiation factor) were used as internal reference genes for quantification. Primers for these genes were designed using Premier 5.0. The reaction conditions for all samples included an initial denaturation step at 95 °C for 2 min, followed by 40 amplification cycles consisting of denaturation at 95 °C for 15 s, annealing and extension at 60 °C for 15 s, and a final denaturation step at 95 °C for 15 s. Each reaction was performed in triplicate. The relative expression level of each individual gene was determined using the comparative cycle threshold (ΔΔCt) method.

## 5. Conclusions

This study systematically characterized fruit development, metabolite accumulation, and tissue-specific carbon allocation in *S. mukorossi*. The fruit reached maturity at approximately 130 DAF and showed four developmental stages: slow growth, rapid expansion, color transition, and maturation. Kernel oil accumulated in a typical S-shaped pattern, whereas pulp saponins increased continuously before maturity and then tended to stabilize. At maturity, soluble sugars were mainly accumulated in the pulp, while starch was relatively enriched in the kernels, indicating distinct carbohydrate storage patterns between the two tissues.

Integrated physiological, metabolomic, and transcriptomic analyses revealed that carbohydrate metabolism plays a central role in supporting the differential accumulation of oil and saponins. In kernels, carbohydrate-derived carbon was mainly associated with fatty acid and oil biosynthesis, accompanied by the preferential expression of lipid biosynthesis-related genes such as *SmACC*, *SmLACS*, *SmGPAT*, and *SmLPAT*. In pulp, carbon was preferentially linked to MVA-dependent triterpenoid saponin biosynthesis and downstream modification processes, with *SmACCT*, *SmMVK*, *SmIDI*, *SmSQE*, and *SmLUP2* identified as important candidate genes. In addition, hormone-related transcription factors and protein interaction networks suggested potential regulatory connections between sugar metabolism and oil or saponin biosynthesis. Overall, these findings indicate that kernel oil and pulp saponin accumulation are supported by distinct but carbohydrate-connected metabolic programs, providing candidate pathways and genes for future functional validation, genetic improvement, and efficient utilization of *S. mukorossi* fruit.

## Figures and Tables

**Table 1:** Comparison of raw and filtered transcriptome assembly data from *S. mukorossi* kernels and pulp.

Sample	Clean Data (%)	Total Mapped Clean Data (%)	Sequenced Refer Genes (%)	Sequenced Novel Genes (%)	Sequenced Total Genes (%)
ZR-S1	45,618,889 (99.47%)	35,963,294 (78.84%)	18,034 (59.98%)	2810 (82.85%)	20,844 (62.97%)
ZR-S2	45,223,768 (99.34%)	38,791,130 (85.79%)	14,778 (49.15%)	1959 (57.78%)	16,738 (50.03%)
ZR-S3	44,978,483 (99.33%)	34,839,474 (77.51%)	14,375 (47.81%)	1691 (49.63%)	16,066 (48.01%)
GP-S1	6,110,223,809 (99.21%)	32,668,126 (79.90%)	16,098 (53.54%)	1717 (45.69%)	17,815 (52.67%)
GP-S2	6,094,369,036 (99.31%)	32,810,904 (80.50%)	15,990 (53.18%)	1719 (45.75%)	17,709 (52.35%)
GP-S3	6,213,349,126 (99.44%)	33,984,466 (81.82%)	15,092 (50.19%)	1394 (37.09%)	16,485 (48.73%)

**Table 2:** qT-PCR gene primers sequence for RNA-seq validation in *S. mukorossi* kernels.

Number	Gene ID	Gene Name	Gene Primers
1	whz_022288	*FabZ*	F: ATTGCCTCACAGGTTTCCGT; R: GAGACCACCAGGCAGGATG
2	whz_006429	*CAC3*	F: ATGTGCAGCGATCTTGTGGA; R: CCTCCAAGCGGTTCAGGAAT
3	whz_005644	*FATA*	F: TGAACCAAGACACGAGACGG; R: TCCGGAAATGCTAACCTCGG
4	whz_006036	*DGAT3*	F: CGATGACGGGTTTCGAAGGA; R: TAACGTTTGGGCCATCACGA
5	whz_006698	*FAD2*	F: GGAGGAGCTTCTTCTTCGTAGG; R: CCTGCACCCATGTTTCTGGAG
6	whz_014788	*TPS12*	F: AGTGAGGTCTGAAGGAAGCTC; R: TCCTTCCCGGCAGTCTGAAT
7	whz_003054	*IPI1*	F: ATTGATGAGGGTGCCCTTGG; R:TCTTCAGCAGGAATGCCCAG
8	whz_025234	*CYP716A15*	F: AGGGCAAGGCCTCACAAAAT; R: GGCCACCAATCAACAAACCG
9	whz_014056	*UGT73C3*	F:AGCTTGAGCCGTGGTACATC; R:CGCGTCCTTATGGAAAAGCG
10	whz_020231	*GAPCP1*	F: TGATGACAACTGTCCACGCA; R: TTTACCTACAGCCTTCGCGG
11	whz_018359	*ATHB-13*	F: AACTCCAAGCTGAGATAATGGG; R: TTTGATGGCGGCGCAGT

## Data Availability

The data presented in this study are available within the article and Supplementary Materials. The *S. mukorossi* kernels and pulp transcriptome raw data can be obtained from the China National Center for Bioinformation (https://ngdc.cncb.ac.cn), GSA number is CRA014727 and CRA033675.

## Supplementary Materials

The following supporting information can be downloaded at: https://www.mdpi.com/article/10.3390/plants15142173/s1.
