# Supplementary material for: Molecular Mechanisms of Oil and Saponin Accumulation and the Regulation of Carbohydrate Metabolism in Sapindus mukorossi Fruit
Source: Plants (Basel). 2026 Jul 15;15(14):2173. doi: 10.3390/plants15142173 (PMC13416213; doi:10.3390/plants15142173)
Supplement: Supplementary file 1 [file plants-15-02173-s001.zip › plants-4354582-Supplementary materials_Figures S1-S4.pdf]

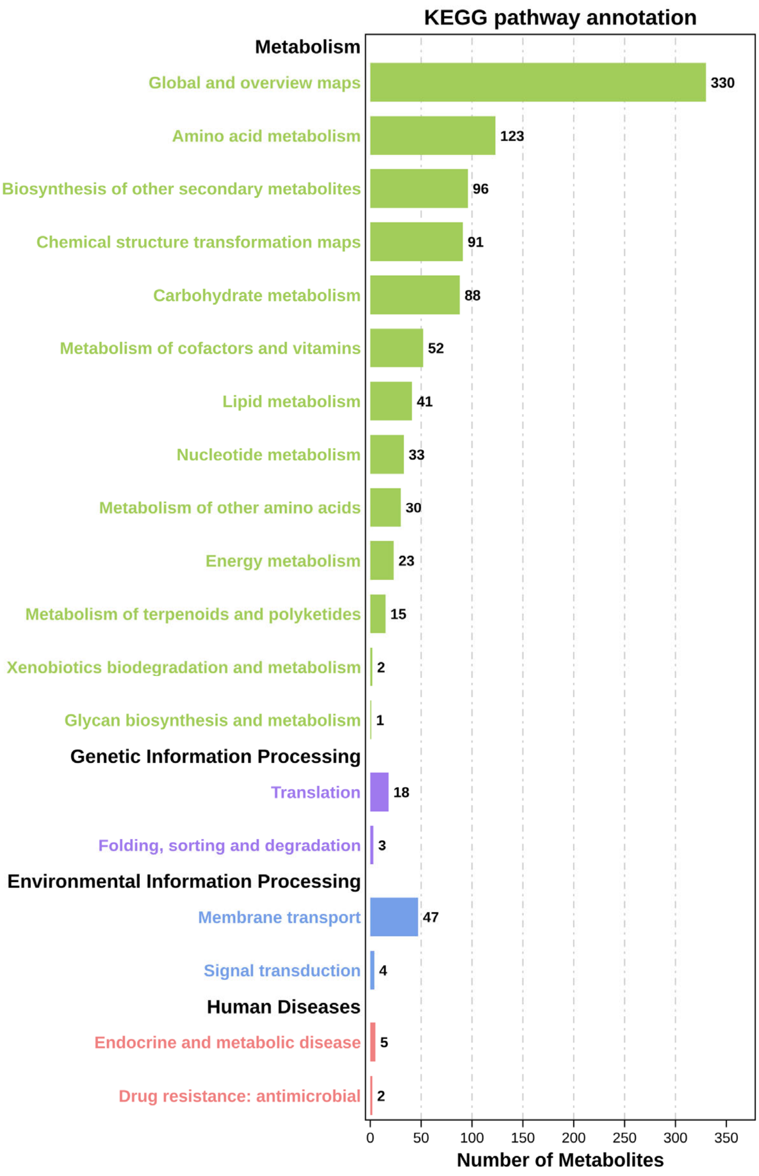

Figure S1. KEGG pathway enrichment of metabolites in *S. mukorossi* pulp

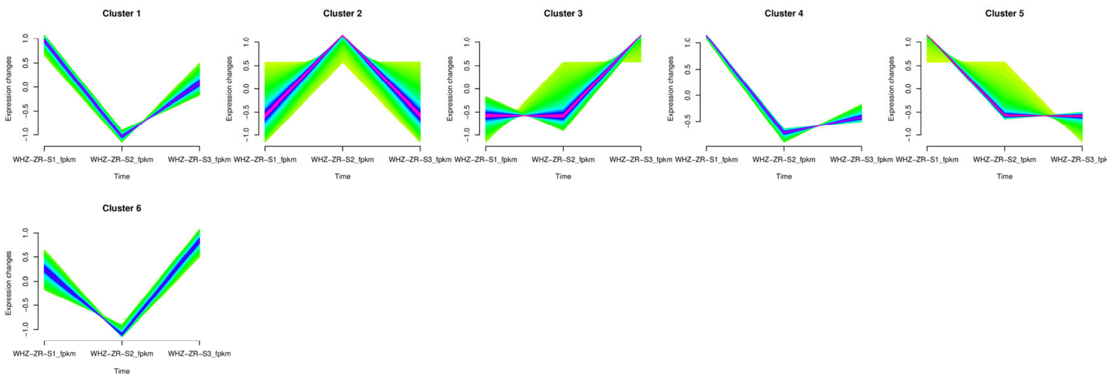

Note: Genes with similar temporal expression patterns across the three developmental stages were grouped into six clusters. The x-axis represents developmental stages, and the y-axis represents normalized expression changes based on FPKM values.

Figure S2. Cluster analysis of transcriptome-wide gene expression profiles in *S. mukorossi* kernels

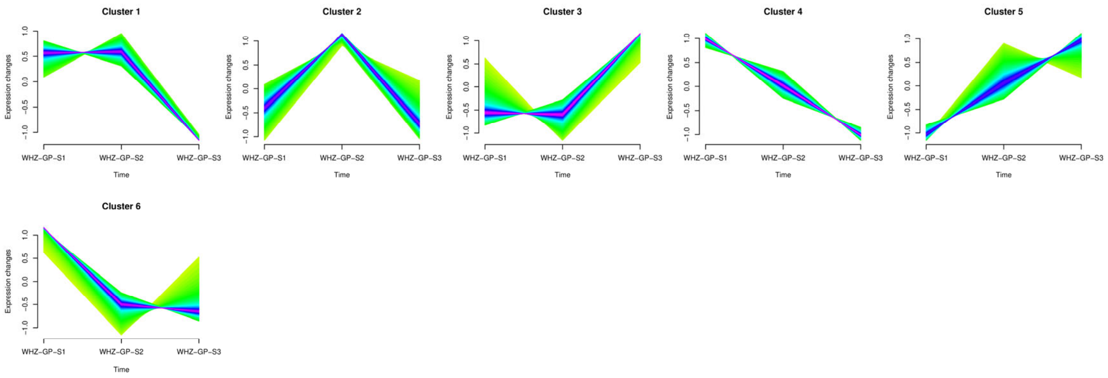

Note: Genes with similar temporal expression patterns across the three developmental stages were grouped into six clusters. The x-axis represents developmental stages, and the y-axis represents normalized expression changes based on FPKM values.

Figure S3. Cluster analysis of transcriptome-wide gene expression profiles in *S. mukorossi* pulp

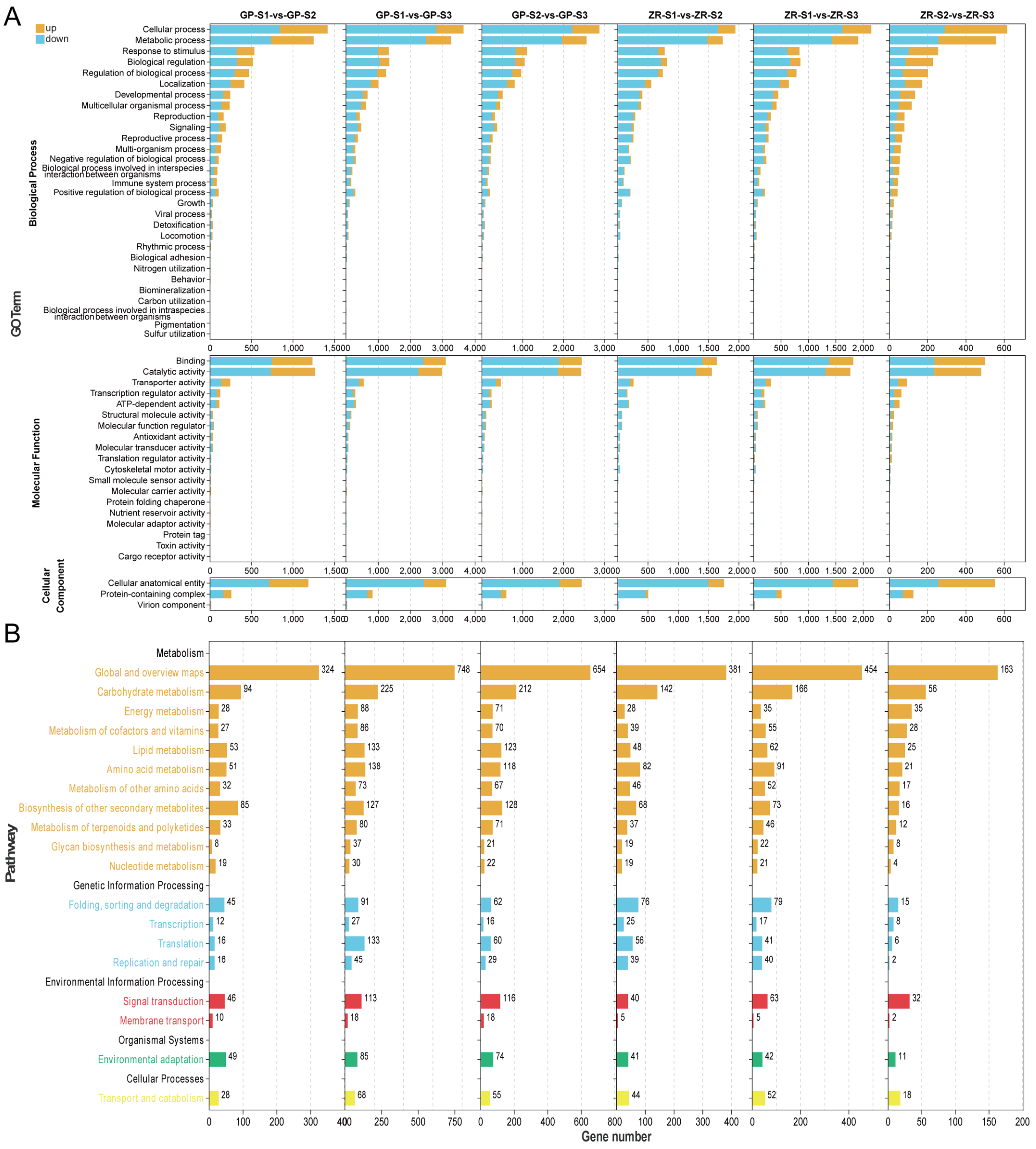

Note: A: GO functional classification. B: KEGG metabolic pathway enrichment.

Figure S4. GO functional annotation and KEGG metabolic pathway enrichment of DEGs in the transcriptome of *S. mukurossi* kernels and pulp
